# Probiotics Evaluation in Oncological Surgery: A Systematic Review of 36 Randomized Controlled Trials Assessing 21 Diverse Formulations

**DOI:** 10.3390/curroncol28060435

**Published:** 2021-12-07

**Authors:** Elise Cogo, Mohamed Elsayed, Vivian Liang, Kieran Cooley, Christilynn Guerin, Athanasios Psihogios, Peter Papadogianis

**Affiliations:** 1 Patterson Institute for Integrative Oncology Research, Canadian College of Naturopathic Medicine, 1255 Sheppard Ave. E., Toronto, ON M2K 1E2, Canada; elisec1234@gmail.com (E.C.); melsayed@ccnm.edu (M.E.); vivian-liang@live.com (V.L.); christilynn.guerin@outlook.com (C.G.); apsihogios@thechi.ca (A.P.); ppnd@outlook.com (P.P.); 2 School of Public Health, Australian Research Centre in Complementary and Integrative Medicine (ARCCIM), University of Technology Sydney, Ultimo 2007, Australia; 3 Pacific College of Health Sciences, San Diego, CA 92108, USA; 4 National Centre for Naturopathic Medicine, Southern Cross University, Lismore 2480, Australia; 5 The Centre for Health Innovation, 429 MacLaren St., Ottawa, ON K2P 0M7, Canada

**Keywords:** cancer, perioperative, synbiotics, prebiotics, *Lactobacillus*, *Bifidobacterium*, nutritional supplements, microbiome, integrative, naturopath

## Abstract

Background: Objectives were to evaluate probiotics safety and efficacy in oncological surgery. Methods: Systematic review methodology guided by Cochrane, PRISMA, SWiM, and CIOMS. Protocol registered on PROSPERO (CRD42018086168). Results: 36 RCTs (on 3305 participants) and 6 nonrandomized/observational studies were included, mainly on digestive system cancers. There was evidence of a beneficial effect on preventing infections, with 70% of RCTs’ (21/30) direction of effect favoring probiotics. However, five RCTs (17%) favored controls for infections, including one trial with RR 1.57 (95% CI: 0.79, 3.12). One RCT that changed (balanced) its antibiotics protocol after enrolling some participants had mortality risk RR 3.55 (95% CI: 0.77, 16.47; 7/64 vs. 2/65 deaths). The RCT identified with the most promising results overall administered an oral formulation of *Lactobacillus acidophilus* LA-5 + *Lactobacillus plantarum* + *Bifidobacterium lactis* BB-12 + *Saccharomyces boulardii*. Methodological quality appraisals revealed an overall substantial risk-of-bias, with only five RCTs judged as low risk-of-bias. Conclusions: This large evidence synthesis found encouraging results from most formulations, though this was contrasted by potential harms from a few others, thus validating the literature that “probiotics” are not homogeneous microorganisms. Given microbiome developments and infections morbidity, further high-quality research is warranted using those promising probiotics identified herein.

## 1. Introduction

The research conducted by the Human Microbiome Project over the past decade, in addition to more recent collective initiatives, estimates that adults host between 1 and 10 times as many bacterial cells as human cells, with most residing in the colon [1]. The intestinal microbiota landscape is a complex ecosystem that is a fundamental biological component and prominently influences human health [2]. The interaction between gastrointestinal microorganisms and the host are complex, forming symbiotic relationships that can both support homeostasis [3], or promote disease development via fortuitous physiologic pathway dysregulation [4]. The microbiome can exert both pro- and anti-inflammatory responses, which may be related to immune functioning, as the immune system can shape the composition of the microbiota [5]. Furthermore, studies have observed that intestinal bacteria may influence oncogenesis, tumor progression, and response to therapy [6]. Postoperative infections are an important factor affecting patient morbidity despite the application of prophylactic therapy with antibiotics and advanced surgical techniques [7]. The rate of developing postoperative infections among patients undergoing abdominal surgery has been estimated to be up to 30%, leading to a significant prolongation of hospital stay, and affecting quality of life [8]. Perioperative management, including antibiotic administration and mechanical bowel preparation, compounded by the physical trauma of surgery can alter the intestinal microbial landscape, intestinal mucosal barrier permeability, and intestinal immune functions [7].

According to a Joint FAO/WHO consensus, probiotics are defined as “live microorganisms which when administered in adequate amounts confer a health benefit on the host” [9]. Modifying the gut microbiota through the use of probiotics has been reported to positively affect various disease states, even in the absence of microbiome alterations. The proposed effects of probiotics and bacteria-colonizing foods may relate to their ability to share genes and metabolites, support challenged microbiota, and directly influence the epithelial and immune cells through modulation of a variety of pathways [10]. In vitro and in vivo studies have demonstrated several different inherent properties of probiotics, including anti-inflammatory, anti-proliferative, and antagonist effects against pathogens [11]. Synbiotics are products that combine probiotics with prebiotics, a specific subset of dietary fibers that are “selectively utilized by host microorganisms conferring a health benefit”, to exert synergistic effect [12]. Synbiotics may reduce pathogenic microorganisms by stimulating the growth of the commensal microbiota and subsequently increasing the production of short chain fatty acids, which are known to stabilize the intestinal barrier and local immune system [13]. Both Lactobacilli (L.) and Bifidobacteria (B.) are lactic acid-producing bacteria that can provide significant health benefits through improving food conversion, growth performance, modulating immune responses and intestinal crypt dynamics, and ultimately protecting against pathogens [14]. Previous systematic reviews in various surgical contexts have found encouraging results from probiotics at reducing postoperative infections and other complications [15]. However, knowledge is accumulating that these effects are likely strain specific [16]. Therefore, even though the literature often categorizes them together, not all probiotic supplements have equivalent safety or efficacy profiles due to their unique properties and effects [17].

While research has shown that the use of probiotics in the general population is generally safe and well tolerated, their application in vulnerable subpopulations requires further consideration of various factors along with careful probiotics selection [18]. The World Gastroenterology Organisation advises that “Most probiotics are designed for the generally healthy population, so use in persons with compromised immune function or underlying severe disease is best restricted to the strains and indications with proven efficacy” [19,20]. Furthermore, patients who are critically ill, hospitalized, immunocompromised, or on broad-spectrum antimicrobials are most at-risk for potentially developing rare adverse effects from probiotics, such as sepsis, fungemia, and gastrointestinal ischemia [21,22].

The complexity and wide array of this heterogeneous and inconsistent literature suggests a need to synthesize and critically appraise the human controlled evidence, with a special focus on evaluating the species and strains studied in diverse formulations. Our objectives were to evaluate the safety and efficacy of adjunctive probiotics use in oncological surgery by conducting an up-to-date, broad, and comprehensive evidence synthesis using rigorous systematic review methodology.

## 2. Materials and Methods

The complete methodology has been described in detail elsewhere [23]. Briefly, the protocol was registered a priori on PROSPERO (CRD42018086168), and followed guidance of the Cochrane Handbook [24], CIOMS Working Group X report [25], and PRISMA reporting criteria [26]. This publication is part of a larger endeavor to prepare multiple systematic reviews based on a prioritization exercise [27], to refine our research agenda and considering 10 natural health products. A detailed report covering the perioperative use of branched-chain amino acids stream was recently published [23]. Study inclusion criteria: studies evaluating synbiotics (i.e., probiotics with prebiotics) or probiotics in patients undergoing cancer-related surgery, any route of administration, duration, dose, and formulation (i.e., used alone or in combinations), compared to active control, placebo, or no added treatment (e.g., standard care). Primary review outcomes included hard endpoints of cancer therapy: cancer treatment response, metastasis/disease progression, mortality, recurrence, remission, and stable disease. Secondary outcomes: anthropometrics (e.g., body weight), bleeding, cancer biomarkers, immune cells, inflammatory marker levels, hospital length of stay, postoperative infections and antibiotics use, other postoperative complications (i.e., ileus/intestinal obstruction/constipation, diarrhea, nausea and vomiting), pain, quality of life, fatigue, and adverse events. Study designs for evaluation of efficacy were limited to randomized controlled trials (RCTs), while for the safety evaluation a broader evidence base was included of controlled observational cohort and case-control studies, nonrandomized and quasi-randomized controlled clinical trials, and RCTs. Studies with postoperative chemotherapy or radiation, non-English reports, and those not reporting probiotic nomenclature were excluded.

The following databases were searched from inception to 19 September 2020: MEDLINE Search Strategy (Supplementary Materials S1), Embase, and Cochrane CENTRAL. The search strategy was peer reviewed by an expert medical librarian (JM) using the Peer Review of Electronic Search Strategies (PRESS) checklist [28], and it is presented in the online Supplementary Materials. Supplemental searches were conducted as per our protocol, and references cited in included studies and related reviews were also scanned. Eligibility screening, data extraction, and risk-of-bias appraisals were done independently in duplicate. The original Cochrane Risk-of-Bias tool [29] was used for RCTs, and the Newcastle-Ottawa Scale [30] was used to assess the methodological quality of nonrandomized and observational studies. Relative risk (RR) for binary outcomes and mean difference (MD) for continuous outcomes were calculated for each trial along with 95% confidence intervals (CIs), when appropriate. Forest plots were created to graphically display data using RevMan 5.4.1, when appropriate [31]. Meta-analysis was not conducted due to the high heterogeneity observed across the very diverse interventions evaluated with respect to different probiotic combinations, genera, species, and strains that each have unique properties and potential effects [17]. Following the Cochrane Handbook and Synthesis Without Meta-analysis (SWiM) guidance, the vote counting of direction of effect method was used for synthesis [24,32]. This method does not provide information about effect size or evaluate statistical significance and is intended to explore trends among studies, so summary results presented across the RCTs necessitate careful interpretation.

## 3. Results

### 3.1. Included Studies

**Figure 1:**
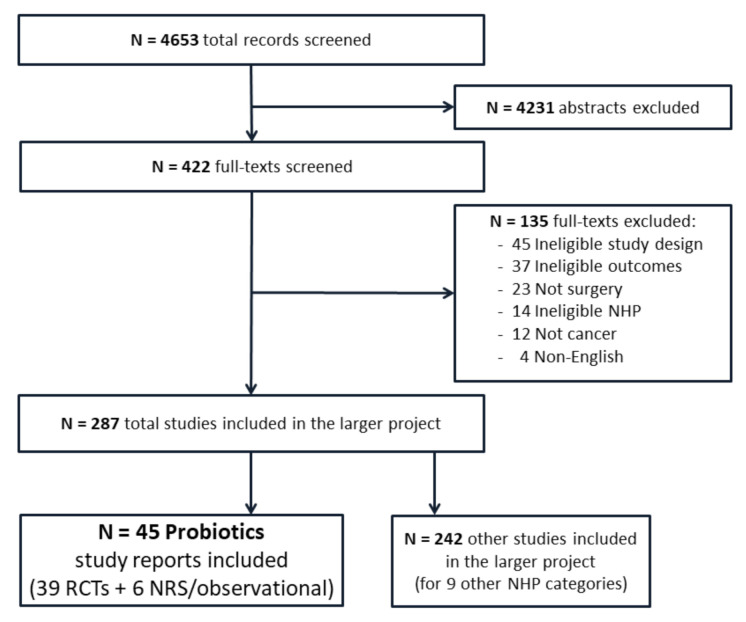
Study flow diagram.

Searches for the overarching umbrella project of 10 natural health products retrieved 4653 records in total, which were screened in duplicate. A total of 45 probiotics articles were included, reporting on 42 unique studies, comprising 39 reports (listed alphabetically under the References) [33,34,35,36,37,38,39,40,41,42,43,44,45,46,47,48,49,50,51,52,53,54,55,56,57,58,59,60,61,62,63,64,65,66,67,68,69,70,71], of 36 randomized controlled trials involving 3305 participants and 6 nonrandomized and cohort studies [72,73,74,75,76,77]. A study flow diagram is presented in Figure 1.

The interventions and comparators evaluated in the 36 included RCTs are presented in Table 1, including dosage and administration details. Supplementary Materials, Table S1 provides the interventions and exposures assessed in the six nonrandomized and observational cohort studies. Table 2 reports the study and patient characteristics of the RCTs; and characteristics of the nonrandomized and observational studies are found in the Supplementary Materials, Table S2. Table S3 provides an overview of the summary characteristics across the 36 RCTs. All except 2 RCTs (94%) dealt with digestive system cancers, and one each was on bladder and head and neck cancers. Roughly half of the RCTs dealt with colorectal cancer (19 RCTs; 53%), followed by hepato-biliary/pancreatic as the second most common cancer types (8 RCTS; 22%). A total of 81% of the included RCTs (29/36) evaluated multi-strain formulations, with a wide variety of bacteria combinations studied. 21 diverse formulations were evaluated, with 42% of the RCTs (15/36) studying unique products (i.e., product included in one RCT). Figure S1 details the six probiotic products and their components that were utilized in more than one RCT. 83% of RCTs (30/36) investigated oral products, while six (16%) administered interventions by enteral nutrition tubes. More than half the RCTs (20/36; 56%) administered the probiotics during both the pre- and post-operative periods, while nine RCTs (25%) utilized them only preoperatively, and in seven RCTs (19%) they were given only postoperatively. A total of 53% of RCTs (19/36) were conducted in Asia, and 31% (11 RCTs) were from Europe. A total of 72% of RCTs were published in the last 10 years (26/36).

### 3.2. Risk-of-Bias Appraisal

**Figure 2:**
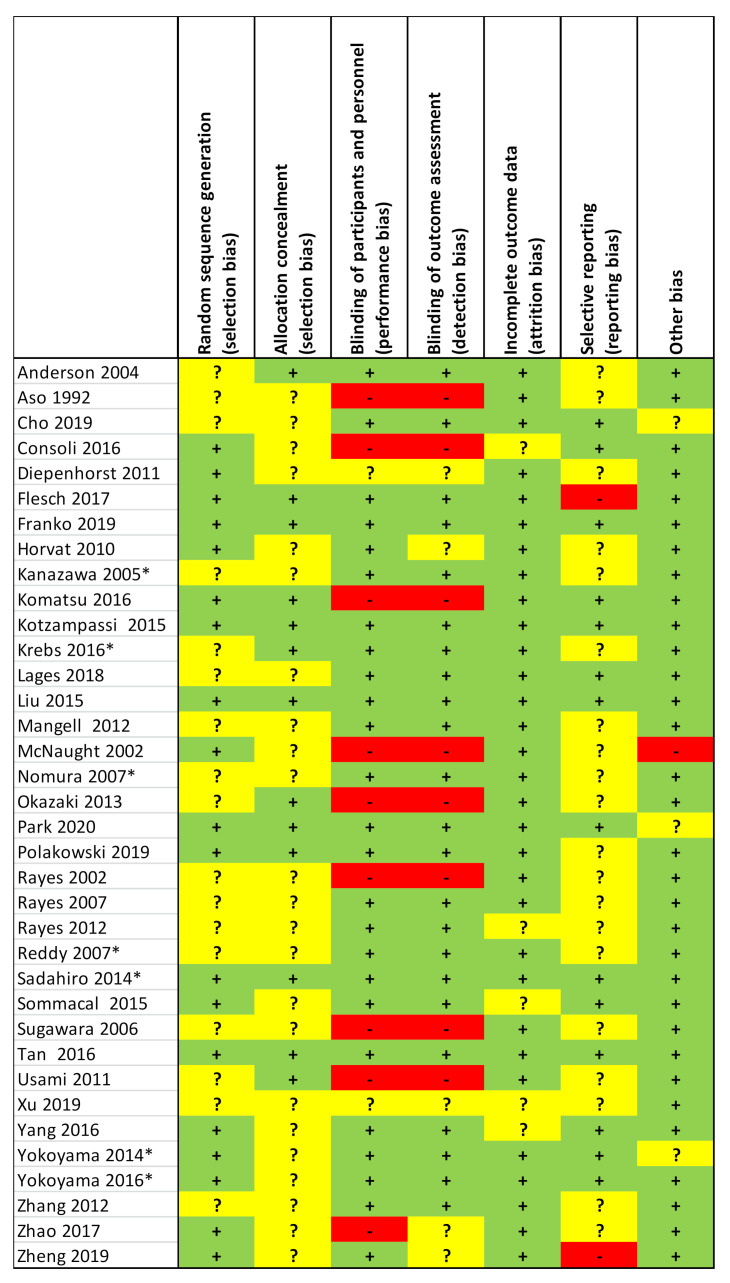
Risk-of-bias appraisal of each RCT. Green “+” = low risk; yellow “?” = unclear risk; red “-” = high risk of bias. * These 7 RCTs had no/unclear blinding but only reported objective outcomes.

**Figure 3:**
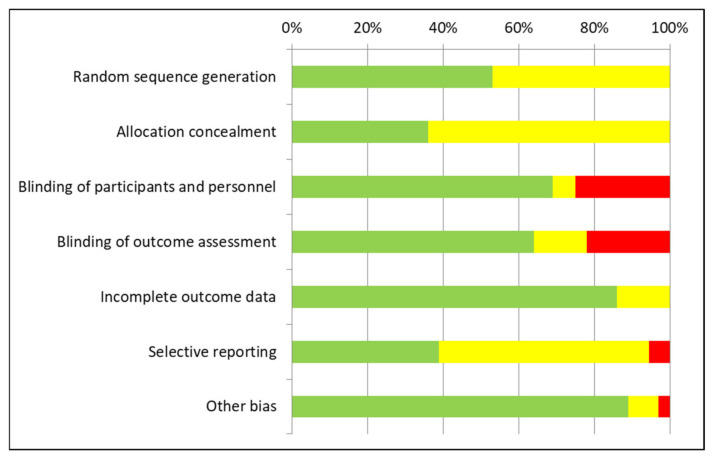
Aggregate risk-of-bias across RCTs. Green = low risk; yellow = unclear risk; red = high risk of bias.

Results for each study of the RCT risk-of-bias assessments using the Cochrane tool are presented in Figure 2, and the aggregate summary results of the 36 RCTs are in Figure 3. A substantial risk-of-bias was found across the majority of RCTs, with only five RCTs (14%) judged as low risk-of-bias on the seven assessment elements in these methodological quality appraisals: Franko et al., 2019; Kotzampassi et al., 2015; Liu et al., 2015; Sadahiro et al., 2014; Tan et al., 2016 [39,43,49,60,63]. Selective reporting (reporting bias) and allocation concealment were the risk-of-bias elements that scored worst. Furthermore, roughly half of the RCTs did not adequately report on random sequence generation, suggesting potential selection bias. Additionally, roughly one-third of RCTs were unclear or high risk for the elements on blinding of participants and personnel and blinded outcome assessment, which suggest potential performance and detection biases.

### 3.3. Mortality and Recurrence

**Figure 4:**
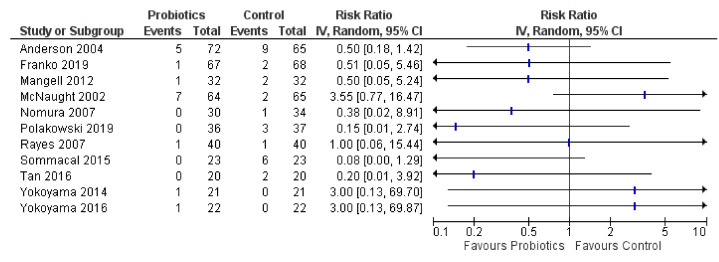
Forest plot for mortality (*n* = 11 RCTs).

The primary outcome of mortality was assessed in 23 RCTs, of which 12 reported that there were no deaths [36,41,42,46,49,56,58,59,62,64,66,69]. Absolute number of events, sample size, and relative risks with 95% CIs for each of the 11 RCTs that reported deaths are presented visually in a forest plot (Figure 4). There was conflicting evidence of any effect on mortality, with 7 of 11 RCTs (64%) favoring probiotics (one had sparse data) based on direction of effect [33,39,50,52,55,61,63], while one (10%) showed no effect [57]. However, three RCTs (20%) favored controls, including the McNaught 2002 trial finding a risk of 30-day mortality of RR 3.55 (95% CI: 0.77, 16.47) in the probiotics group (7/64; 11%) vs. controls (2/65; 3%). Of note, however, this trial amended its protocol after 11 probiotics patients had already been enrolled to also give the probiotics arm antibiotic prophylaxis (the initial comparison was intended to be probiotics vs. antibiotics), yet mortality data was only reported including these 11 patients who had not received antibiotics preoperatively. This trial administered *Lactobacillus plantarum* 299v + oatmeal to patients undergoing colorectal and abdominal surgeries [51]. The other 2 RCTs by Yokoyama et al., 2014 and Yokoyama et al., 2016 had sparse data (i.e., only one death in total occurred in each trial) [67,68]. An additional RCT presented results graphically for alive hospital discharges at 31 days showing almost all participants in the probiotics group compared to roughly 10% fewer in controls [43].

Only one RCT reported on cancer recurrence. Assessed in superficial bladder cancer surgery, the probiotics group had a 1.8-times-longer 50% recurrence-free interval than controls (*p* = 0.03) [34]. None of the RCTs reported data on the other primary outcomes of our review (i.e., hard treatment endpoints).

### 3.4. Postoperative Infections

**Figure 5:**
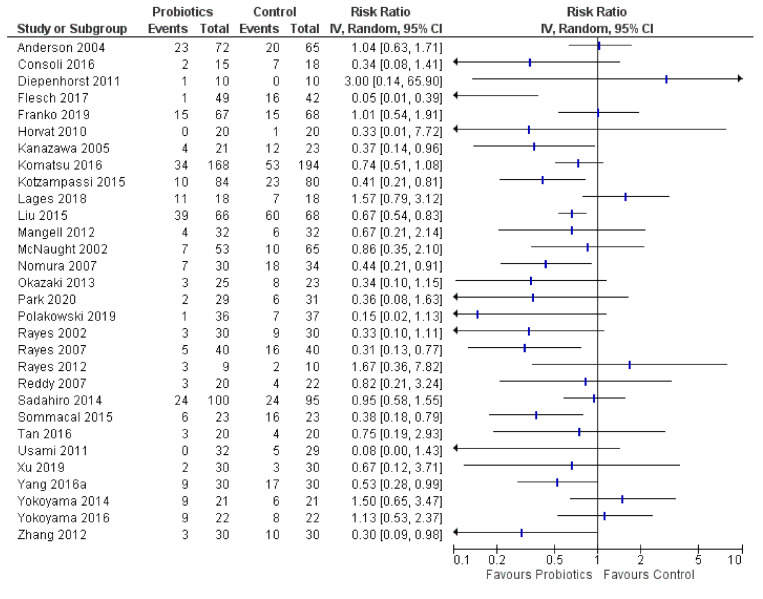
Forest plot for infections (*n* = 30 RCTs).

Thirty RCTs reported on total postoperative infections, which are presented in a forest plot (Figure 5). Across these studies, there was evidence based on direction of effect that probiotics had a beneficial effect on postoperative infections, with 21 of 30 RCTs (70%) favoring them (one had sparse data), while another four (13%) showed no effect. In contrast, five RCTs (17%) favored controls [37,46,58,67,68]. The Lages et al., 2018 trial found a postoperative infection risk of RR 1.57 (95% CI: 0.79, 3.12) in the probiotics group vs. controls (11/18 vs. 7/18), and this trial in head and neck cancers administered *Lactobacillus acidophilus* NCFM + *L. rhamnosus* HN00l + *L. paracasei* LPC-31 + *Bifidobacterium lactis* HN019 + fructooligosaccharides [46]. Yokoyama et al., 2014 found a RR 1.50 (95% CI: 0.65, 3.47) [67]. The other three negative RCTs had wide confidence intervals (one had sparse data).

In addition, bacteremia/sepsis data were evaluated for safety as a specific subgroup of severe postoperative infectious complications, which were reported on in 12 of the above RCTs (Table S4). This revealed that two RCTs (17%) found higher rates in the probiotics group compared to controls. Yokoyama et al., 2014 had two cases (RR 5.00; 95% CI: 0.25, 98.27; 2/21 vs. 0/21) [67]; and another trial had one additional central line infection (RR 1.20; 95% CI: 0.43, 3.39; 7/66 vs. 6/68) [49]. Furthermore, since most studies were found to be on gastrointestinal cancer surgeries, a post-hoc analysis is presented on the RCTs that specifically reported a breakdown of anastomotic leakage/abdominal abscess subgroup type of infections (Table S4). Only 1 out of 16 (6%) RCTs (Yokoyama et al., 2014 [67]) found a higher rate of these events in the probiotics compared to control groups.

**Figure 6:**
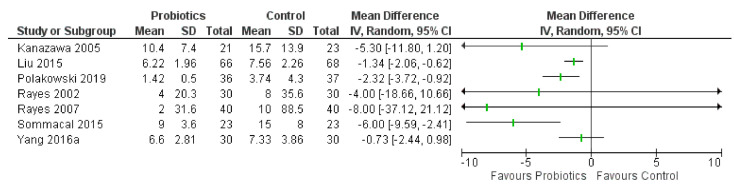
Forest plot for antibiotics duration of use, in days (*n* = 7 RCTs).

Corresponding to the infections results, there was evidence that probiotics had a beneficial effect on the length of antibiotic therapy, with seven of seven RCTs (100%) favoring them. All seven studies are included in the infections outcome above. Figure 6 presents a forest plot of the mean difference in days for each RCT.

### 3.5. Ileus and Intestinal Obstruction

**Figure 7:**
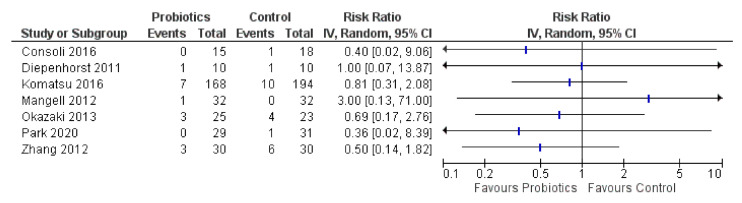
Forest plot for ileus or intestinal obstruction (*n* = 7 RCTs).

Postoperative ileus or intestinal obstruction comparative results were evaluated in seven RCTs (forest plot details presented in Figure 7). Evidence was unclear for any effect of probiotics on ileus or intestinal obstruction. Four of seven RCTs (57%) favored probiotics (two had sparse data), while one (14%) with sparse data favored controls (one event total) [50], and two RCTs (29%) showed no effect.

### 3.6. Diarrhea

**Figure 8:**
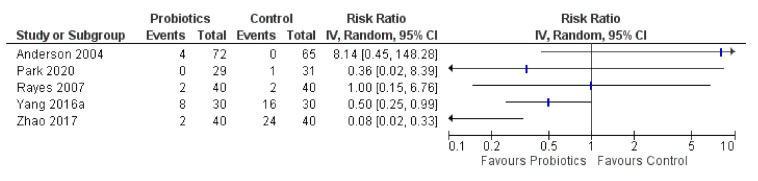
Forest plot for diarrhea (*n* = 5 RCTs).

Comparative results on diarrhea were assessed in seven RCTs, of which two had no cases [37,56]. There was conflicting evidence of any effect on diarrhea, as three of five RCTs (60%) favored probiotics (one had sparse data), while one (20%) showed no effect (forest plot in Figure 8). However, one RCT (20%) by Anderson et al., 2004 favored controls, with an increased risk of diarrhea of RR 8.14 (95% CI: 0.45, 148.28) for probiotics (4/72; 6%) vs. controls (0/65). This trial dealt with colon cancer and other abdominal surgeries and used a combination of *Lactobacillus acidophilus* La5 + *L. bulgaricus* + *Bifidobacterium lactis* Bb-12 + *Streptococcus thermophilus* + oligofructose [33].

### 3.7. White Blood Cells and C-Reactive Protein

Leukocyte counts were assessed in 12 RCTs. Details are reported in Supplementary Materials, Table S4. The evidence was unclear for any effect on total white blood cells (WBCs) as seven RCTs (58%) showed no effect and three (25%) favored probiotics, while two RCTs (17%) [67,68], favored controls. Similarly, the effect was unclear for lymphocytes reported on in six RCTs, with three RCTs (50%) showing no effect and two (33%) favoring probiotics, while one RCT (17%) [56], favored controls (Table S4). Neutrophil counts were reported on in only three RCTs, and no effect was seen from probiotics compared to baseline. The evidence was also unclear for any effect on C-reactive protein levels reported on in 13 RCTs, with six of them showing no effect and three favored probiotics, whereas four RCTs (31%) [33,40,51,68] favored controls (Table S4).

### 3.8. Hospital Length of Stay

Duration of hospitalization details are presented for 22 RCTs in Table S4. 19 of these studies are also included in the infections outcome (Figure 5), which is a major contributing factor for length of stay. For the other three RCTs, the number of days in hospital was fairly similar between groups [45,68,70].

### 3.9. Additional Outcomes

Eleven RCTs utilizing probiotics preoperatively reported on blood loss (Table S4). Two (18%) RCTs found more blood loss in the probiotics groups; however, in both trials, mean operative times were 1 h longer and they reported more extensive resection/dissection occurred in the probiotics groups [64,67]. Pain was evaluated in seven RCTs (six on abdominal pain/cramps and one reporting pain scores). The evidence was unclear for any effect on pain with five RCTs showing no effect and two favored probiotics (Table S4). Two RCTs reported comparative results between groups on nausea/vomiting; one found a greater increase on a nausea score (FACT-G7 subscale) in the probiotics group vs. placebo [39], while the other had fewer cases in the synbiotics arm compared to fiber-free enteral nutrition (0/40 vs. 4/40) [70]. Quality of life was assessed in only one RCT using the FACT-G7 scale, and it found that reduction of quality of life was mitigated in the probiotics group [39]. This same RCT also reported FACT-G7 subscale results for fatigue, with a smaller increase in fatigue in the probiotics group [39]. One RCT reported on anthropometrics, finding mean weight loss was similar between groups (2.0 vs. 2.2 kg for synbiotics and controls) [56]. Cancer biomarker secondary outcomes were not reported.

### 3.10. Adverse Events in RCTs

Adverse events in RCTs are presented in detail in Table S5. Roughly half of the RCTs (17/36; 47%) did not report data about side-effects. Nine (25%) of the RCTs reported that none of the participants experienced an adverse event due to the intervention. Another 10 (28%) RCTs reported there were no severe adverse events due to the intervention, with the most common side-effects of the interventions being nausea and flatulence. One RCT reported that “we could not exclude the relationship of 2 adverse events with the test powder in the probiotics group (cholelithiasis and tremor) and 3 adverse events in the placebo group (diarrhea, tremor, and abnormal liver function)”. This trial in sigmoid colon cancer utilized *Bifidobacterium animalis* subsp. *lactis* HY8002 + *Lactobacillus casei* HY2782 + *L. plantarum* HY7712 + xylooligosaccharides + fructooligosaccharides [54]. Other postoperative complications not captured above are also presented for additional safety data (Table S5). Total noninfectious complications were in general either similar or lower in the probiotics groups. However, two (6%) RCTs found a higher rate each of pancreatic fistula (≥grade B) [68], and 30-day readmission [39], in probiotics arms.

### 3.11. Nonrandomized and Observational Studies

The methodological quality appraisals with the Newcastle-Ottawa Scale of the 3 nonrandomized and 3 observational cohort studies rated them as low methodological quality (Table 3). In particular, there was a lack of controlling for important potential confounding in the “comparability of cohorts on the basis of the design or analysis” item, which is a key issue for nonrandomized and observational studies. Detailed results from all six of these studies are reported in Table S6. For the primary review outcomes, only two studies reported on mortality and one on disease progression and there was no evidence of a safety concern for these outcomes from these studies. Infections were reported in three studies, with two showing no effect and one finding more enteritis (5/75 vs. 3/81) yet fewer surgical site infections (7/75 vs. 20/81) in the probiotics group [72]. Additionally, 1 study found more patients in the probiotics group (5/23 vs. 2/22) required additional antibiotics [76]. two studies reported on length of stay, one on pain, and three on blood loss, with no evidence of a safety concern for these outcomes. Regarding adverse events and other complications not captured above, three studies did not report on adverse events [72,73,75], one found fewer complications in the probiotics group, and one found an additional serious complication (Clavien-Dindo Grade IIIb or IV) in the probiotics group (2/42 vs. 1/55) [74].

## 4. Discussion

This large and up-to-date evidence synthesis presents the most comprehensive systematic review on this topic including 36 RCTs in 3305 participants plus six nonrandomized/observational cohort studies. Uniquely, 21 diverse probiotic formulations were evaluated across these RCTs, thereby providing a novel assessment from human controlled studies of the evidence profiles of various probiotics genera, species, strains, and combinations in this oncological surgery population. 94% of RCTs dealt with digestive system cancers, with 53% on colorectal cancer (19/36) and 22% on hepato-biliary/pancreatic cancers. 83% investigated oral products, and six RCTs administered probiotics in enteral nutrition tubes. Our findings support current thinking that probiotics effects are specific to the product/formulation. Due to substantial heterogeneity among interventions, overall conclusions regarding “probiotics” in general cannot be made, and thus we did not pool results in a meta-analysis. From our analysis, the placebo-controlled RCT identified with the most promising results overall based on direction of effect from among five trials judged as low risk-of-bias was Kotzampassi et al., 2015. This RCT in colorectal cancer included 164 total participants and gave an oral formulation of *Lactobacillus acidophilus* LA-5 (1.75 billion CFU) + *L. plantarum* (0.5 billion CFU) + *Bifidobacterium lactis* BB-12 (1.75 billion CFU) + *Saccharomyces boulardii* (1.5 billion CFU) twice a day from 1 day pre-op until 14 days post-op [43].

There was promising evidence that probiotics may have a beneficial effect on postoperative infections with 21 of 30 RCTs (70%) favoring them compared to controls based on direction of effect. In contrast, 17% (5/30) favored controls, including the Lages 2018 trial finding a postoperative infection risk of RR 1.57 (95% CI: 0.79, 3.12) with a combination of *Lactobacillus acidophilus* NCFM + *L. rhamnosus* HN00l + *L. paracasei* LPC-31 + *Bifidobacterium lactis* HN019 + fructooligosaccharides [46]. One safety concern identified in one RCT out of 11 (9%) found a greater risk of mortality (RR 3.55; 95% CI: 0.77, 16.47) in the probiotics group; however, upon inspection they did not give 11 probiotics patients (17%) antibiotic prophylaxis and later amended their protocol (whereas the initial intended comparison was probiotics vs. antibiotics). This RCT administered *Lactobacillus plantarum* 299v + oatmeal to patients undergoing colorectal and other abdominal surgeries [51]. This finding conflicted with 7 RCTs (64%) that favored probiotics regarding mortality. Conflicting evidence was also found for diarrhea, with three of five RCTs (60%) favoring probiotics, one showing no effect, and one favoring controls. The latter trial found an increased risk of diarrhea of RR 8.14 (95% CI: 0.45, 148.28) in colon cancer and other abdominal surgery patients administering a combination of *Lactobacillus acidophilus* La5 + *L. bulgaricus* + *Bifidobacterium lactis* Bb-12 + *Streptococcus thermophilus* + oligofructose [33].

Regarding adverse events, there wasn’t evidence of a difference in types and rates reported between groups overall. However, adverse events were poorly reported with 47% of RCTs not reporting about side-effects. A total of 25% of RCTs (9/36) reported that none of the participants experienced an adverse event due to the intervention, and 28% reported there were no severe adverse events due to the intervention. The most common side-effects of probiotics or synbiotics were nausea and flatulence. Among the six nonrandomized and observational studies, there was no evidence of a safety concern. These six studies were of low methodological quality, especially due to lack of controlling for confounding.

There was overall substantial risk-of-bias across most studies since only 14% of RCTs (5/36) were judged as low risk-of-bias in the seven elements of the Cochrane tool. In particular, there was potential selection bias and reporting bias as random sequence generation and allocation concealment were often not adequately reported, and many RCTs did not cite a trial registration record or accessible protocol.

Several related systematic reviews have been published on probiotics [11,15,78,79,80,81,82,83,84,85]. In addition to uniquely differentiating between 21 formulations, our systematic review includes more RCTs, is up to date, includes all cancer types, evaluates a broader range of outcomes, and it focuses on the oncological perioperative period. Four other systematic reviews also investigated mortality, and overall, they found no significant differences between groups [80,82,84,85]. Consistent with our findings, a beneficial effect of probiotics on preventing postop infections was found in several systematic reviews and meta-analyses in colorectal cancer patients [(odds ratio, OR 0.34; 95% CI: 0.21, 0.54) [78], (*p* < 0.05) [11], (OR 0.51; 95% CI: 0.38, 0.68) [79], (RR 0.72; 95% CI: 0.56, 0.92) [15], (OR 0.28; 95% CI: 0.20, 0.39) [81]], and also in non-cancer (mainly gastrointestinal) surgical populations [(RR 0.55; 95% CI: 0.39, 0.78) [83], (RR 0.48; 95% CI: 0.36, 0.63) [80], (RR 0.56; 95% CI: 0.46, 0.69) [84], (OR 0.35; 95% CI: 0.24, 0.50) [85], (RR 0.26; 95% CI: 0.08, 0.84) [82]]. Beneficial effects of probiotics on length of antibiotic therapy and/or hospital length of stay were also found in other systematic reviews [11,15,78,80,81,83,84,85]. Regarding safety, one systematic review reported that intake was well tolerated overall, and that rates of abdominal distension, cramps, and diarrhea were not significantly elevated compared to controls in the perioperative setting [84], while two reported fewer side effects from perioperative probiotics use [78,80]. Most of the systematic reviews reported substantial heterogeneity among the different probiotics ingredients evaluated; thus, conclusions on the best regimens, strains, dosages, and durations of use were not reported [11,15,78,79,80,83,84]. One systematic review found no significant differences in infections comparing multi-strain vs. single strain probiotics, or when preoperative administration was compared to both pre- and postoperative use [81].

This systematic review has several strengths. Protocol details were registered a priori online on the PROSPERO registry, the literature search was conducted for comprehensiveness and peer reviewed, studies were assessed for risk-of-bias, there was a broad range of outcomes evaluated, and methods and results were reported according to guidelines. The main limitations at the review-level were including only English-language reports and not searching databases from Asia (potentially adding language bias), and the direction of effect synthesis method. The main study-level limitation of the results was the majority of RCTs being judged as high/unclear risk-of-bias, which weakens the findings. Also, many studies reported adverse events inadequately. Not surprisingly, there was a high degree of heterogeneity among the 21 formulations of probiotics. Finally, some trials did not report the ‘strain’ used of the probiotics.

## 5. Conclusions

This large systematic review evaluated 21 diverse formulations and found encouraging results with several probiotics in this specific patient population. It also highlighted potential harms from others, thereby emphasizing the importance of not grouping all probiotics into one general category due to differing effects. Given recent developments about microbiome and the morbidity from postoperative infections, further high-quality research is warranted using those promising probiotics identified herein.

## Figures and Tables

**Table 1:** Interventions and comparators evaluated in 36 included RCTs.

Author Year.	Interventions and Comparators with Dosages	Freq. of Dose	Route of Admin.	Tx Duration Pre-op (Days)	Tx Duration Post-op (Days)	Tx Duration TOTAL (Days)
Anderson 2004 [33]	*Lactobacillus acidophilus* La5 + *L. bulgaricus* + *Bifidobacterium lactis* Bb-12 + *Streptococcus thermophilus* (4 billion CFU) capsule + oligofructose (11 g) powder ^a^	TID	oral	12	4	16
	Placebo capsule + sucrose placebo powder	TID	oral	12	4	16
Aso 1992 [34]	*Lactobacillus casei* (10 billion viable cells)	TID	oral	NA	365	365
	Standard care alone	NA	NA			
Cho 2019 [35]	*Lactobacillus plantarum* CJLP243 (10 billion)	QD	oral	1	21	22
	Maltodextrin + glucose placebo (2 g)	QD	oral	1	21	22
Consoli 2016 [36]	*Saccharomyces boulardii* (50 million CFU)	QD	oral	9	NA	9
	Standard care alone	NA	NA			
Diepenhorst 2011 [37]	*Bifidobacterium bifidum* + *B. infantis* + *Lactobacillus acidophilus* + *L. casei* + *L. salivarius* + *L. lactis* (10 billion)	BID	oral	7	7	14
	Standard tx control ^b^ [neither probiotics nor SDD]	NR	NA	NR	NR	NR
	Selective decontamination of the digestive tract (SDD antibiotics regimen)	4 times daily	Multiple	4	2	6
Flesch 2017 [38]	*Lactobacillus acidophilus* NCFM + *L. rhamnosus* HN001 + *L. paracasei* LPC-37 + *Bifidobacterium lactis* HN019 (1 billion CFU each) + fructooligosaccharides ^a^ (6 g)	BID	oral	5	14	19
	Maltodextrin placebo (6 g)	BID	oral	5	14	19
Franko 2019 [39]	*Bifidobacterium breve* + *B. longum* + *B. infantis* + *Lactobacillus acidophilus* + *L. plantarum* + *L. paracasei* + *L. bulgaricus* + *Streptococcus thermophilus* (112.5 billion CFU/capsule)	BID	oral	1	6	7
	Placebo	BID	oral	1	6	7
Horvat 2010 [40]	*Pediacoccus pentosaceus* 5–33:3 + *Leuconostoc mesenteroides* 32–77:1 + *Lactobacillus paracasei* subsp. *paracasei* 19 + *L. plantarum* 2362 (10 billion each) + betaglucan + inulin + pectin + resistant starch fibers ^a^ (2.5 g each) [without mechanical bowel preparation]	BID	oral	3	NA	3
	Mechanical bowel preparation control ^b^	QD	oral	1	NA	1
	Heat-inactivated lactobacilli + betaglucan + inulin + pectin + resistant starch fibers (2.5 g each) [without mechanical bowel preparation]	BID	oral	3	NA	3
Kanazawa 2005 [41]	*Lactobacillus casei* strain Shirota (300 million) + *Bifidobacterium breve* strain Yakult (300 million) + galactooligosaccharides ^a^ (12 g) + EN + PN	QD	enteral	NA	14	14
	Standard EN + PN	QD	NA	NA	14	14
Komatsu 2016 [42]	*Lactobacillus casei* strain Shirota (40 billion) + *Bifidobacterium breve* strain Yakult (10 billion) + galactooligosaccharides ^a^ (2.5 g)	QD	oral	7–11	Yes	Total NR
	Standard care alone	NA	NA			
Kotzampassi 2015 [43]	*Lactobacillus acidophilus* LA-5 (1.75 billion CFU) + *L. plantarum* (0.5 billion CFU) + *Bifidobacterium lactis* BB-12 (1.75 billion CFU) + *Saccharomyces boulardii* (1.5 billion CFU)	BID	oral	1	15	16
	Glucose polymer placebo	BID	oral	1	15	16
Krebs 2016 [45]	*Pediacoccus pentosaceus* 5–33:3 + *Leuconostoc mesenteroides* 32–77:1 + *Lactobacillus paracasei* subsp. *paracasei* 19 + *L. plantarum* 2362 (100 billion each) + betaglucan + inulin + pectin + resistant starch fibers ^a^ (2.5 g each) [without mechanical bowel preparation]	BID	oral	3	NA	3
	Mechanical bowel preparation control	QD	oral	1	NA	1
	Betaglucan + inulin + pectin + resistant starch fibers (2.5 g each) [without mechanical bowel preparation]	BID	oral	3	NA	3
Lages 2018 [46]	*Lactobacillus paracasei* LPC-31 + *L. rhamnosus* HN00l + *L. acidophilus* NCFM + *Bifidobacterium lactis* HN019 (1 billion CFU/mL each) + fructooligosaccharides ^a^ (6 g) diluted in 20 mL of water + standard EN	BID	enteral	NA	5–7	5–7
	Maltodextrin placebo (6 g) + standard EN	BID	enteral	NA	5–7	5–7
Liu 2015 [49]	*Lactobacillus plantarum* CGMCC No. 1258 (200 billion CFU) + *L. acidophilus* LA-11 (140 billion CFU) + *Bifidobacterium longum* BL-88 (100 billion CFU)	QD	oral	6	10	16
	Maltodextrin placebo	QD	oral	6	10	16
Mangell 2012 [50]	*Lactobacillus plantarum* 299v (100 billion CFU) in an oatmeal-based drink ^a^ (100 mL)	QD	oral	8	5	13
	Oatmeal-based placebo drink without probiotics (100 mL)	QD	oral	8	5	13
McNaught 2002 [51]	*Lactobacillus plantarum* 299v (25 billion CFU/day) in an oatmeal-based drink ^a^	NR	oral	IQR: 7–12	IQR: 4–9	med.: 14
	Standard care alone	NA	NA			
Nomura 2007 [52]	*Enterococcus fecalis* T-110 (12 mg/day) + *Clostridium butyricum* TO-A (60 mg/day) + *Bacillus mesentericus* TO-A (60 mg/day)	NR	oral	3–15	9–38	12–53
	Standard care alone	NA	NA			
Okazaki 2013 [53]	*Lactobacillus casei* strain Shirota (1 g) + *Bifidobacterium breve* strain Yakult (1 g) + galactooligosaccharides ^a^ (15 g)	QD	oral	7	10	17
	Standard care alone	NA	NA			
Park 2020 [54]	*Bifidobacterium animalis* subsp. *lactis* HY8002 (100 million CFU) + *Lactobacillus casei* HY2782 (50 million CFU) + *L. plantarum* HY7712 (50 million CFU) + xylooligosaccharides (350 mg) + fructooligosaccharides (36 mg)	BID	oral	7	21	28
	Xylooligosaccharides (350 mg) + fructooligosaccharides (36 mg)	BID	oral	7	21	28
Polakowski 2019 [55]	*Lactobacillus acidophilus* NCFM + *L. rhamnosus* HN001 + *L. paracasei* LPC-37 + *Bifidobacterium lactis* HN019 (1 billion each) + fructooligosaccharides ^a^ (6 g) ^c^	BID	oral	7	NA	7
	Maltodextrin placebo	BID	oral	7	NA	7
Rayes 2002 [56]	Live *Lactobacillus plantarum* 299 (1 billion) + oat fibe r ^a^ (11.3 g/L) + EN	BID	enteral	NA	4–5	4–5
	Standard total parenteral nutrition or fiber-free EN control ^b^	QD	IV	NA	8	8
	Heat-killed *Lactobacillus plantarum* 299 + oat fiber (11.3 g/L) + EN	QD	enteral	NA	4–5	4–5
Rayes 2007 [57]	*Pediacoccus pentosaceus* 5–33:3 + *Leuconostoc mesenteroides* 32–77:1 + *Lactobacillus paracasei* subsp. *paracasei* 19 + *L. plantarum* 2362 (10 billion) + betaglucan + inulin + pectin + resistant starch fibers ^a^ (2.5 g each) + EN	BID	enteral	1	8	9
	Betaglucan + inulin + pectin + resistant starch fibers (2.5 g each) + EN	BID	enteral	1	8	9
Rayes 2012 [58]	*Pediacoccus pentosaceus* 5–33:3 + *Leuconostoc mesenteroides* 32–77:1 + *Lactobacillus paracasei* subsp. *paracasei* 19 + *L. plantarum* 2362 (10 billion) + betaglucan + inulin + pectin + resistant starch fibers ^a^ (2.5 g each) + EN	BID	enteral	1	10	11
	Betaglucan + inulin + pectin + resistant starch fibers (2.5 g each) + EN	BID	enteral	1	10	11
Reddy 2007 [59]	*Lactobacillus acidophilus* La5 + *L. bulgaricus* + *Bifidobacterium lactis* Bb-12 + *Streptococcus thermophilus* (4 billion CFU) + oligofructose ^a^ (10 g) + neomycin (3 g) + mechanical bowel preparation	TID	oral	NR	NA	NR
	Neomycin (3 g) + mechanical bowel preparation control ^b^	TID	oral	NR	NA	NR
	*Lactobacillus acidophilus* La5 + *L. bulgaricus* + *Bifidobacterium lactis* Bb-12 + *Streptococcus thermophilus* (4 billion CFU) + oligofructose (10 g) + neomycin (3 g) [without mechanical bowel preparation]	TID	oral	NR	NA	NR
	Mechanical bowel preparation only	QD	oral	1	NA	1
Sadahiro 2014 [60]	*Bifidobacterium bifidum* (3.3 billion) + maltooligosaccharide [plus single IV dose of flomoxef; & standard mechanical bowel preparation]	TID	oral	7	10	17
	Standard care alone control ^b^ [plus single IV dose of flomoxef; & standard mechanical bowel preparation. No probiotic or oral antibiotics]	NA	NA			
	Kanamycin sulfate + metronidazole (500 mg each) [plus single IV dose of flomoxef; & standard mechanical bowel preparation]	TID	oral	1	NA	1
Sommacal 2015 [61]	*Lactobacillus acidophilus* 10 + *L. rhamnosus* HS 111 + *L. casei* 10 + *Bifidobacterium bifidum* (1 billion CFU each) + fructooligosaccharides (100 mg) ^d^	BID	oral	4	10	14
	Sucrose placebo	BID	oral	4	10	14
Sugawara 2006 [62]	Pre-op: Oral *Lactobacillus casei* strain Shirota (40 billion) + *Bifidobacterium breve* strain Yakult (10 billion) + galactooligosaccharides ^a^ (15 g). Post-op: *Lactobacillus casei* strain Shirota (300 million) + *Bifidobacterium breve* strain Yakult (300 million) + galactooligosaccharides ^a^ (15 g) + standard EN + PN.	QD	oral & enteral	14	14	28
	Pre-op: Standard care alone. Post-op: *Lactobacillus casei* strain Shirota (300 million) + *Bifidobacterium breve* strain Yakult (300 million) + galactooligosaccharides ^a^ (15 g) + standard EN + PN.	QD	enteral	NA	14	14
Tan 2016 [63]	*Lactobacillus acidophilus* BCMC12130 + *L. casei* BCMC12313 + *L. lactis* BCMC12451 + *Bifidobacterium bifidum* BCMC02290 + *B. longum* BCMC02120 + *B. infantis* BCMC02129 (30 billion CFU)	BID	oral	7	NA	7
	Placebo (3 g)	BID	oral	7	NA	7
Usami 2011 [64]	*Lactobacillus casei* strain Shirota (300 million) + *Bifidobacterium breve* strain Yakult (300 million) + galactooligosaccharides ^a^ (10 g) [+ PN for 4 days post-op]	QD	oral	14	12	26
	Standard care alone [+ PN for 4 days post-op]	NA	NA			
Xu 2019 [65]	Bifidus-triple viable preparation ^e^ + glucose solution	QD	oral	7	NA	7
	Glucose solution	QD	oral	7	NA	7
Yang 2016 [66]	*Bifidobacterium longum* + *Lactobacillus acidophilus* + *Enterococcus faecalis* ^a^ (20 million CFU each)	TID	oral	5	7	12
	Maltodextrin + sucrose placebo (2 g)	TID	oral	5	7	12
Yokoyama 2014 [67]	Pre-op: Oral or enteral *Lactobacillus casei* strain Shirota (40 billion) + *Bifidobacterium breve* strain Yakult (10 billion) + galactooligosaccharides ^a^ (15 g). Post-op: Enteral *Lactobacillus casei* strain Shirota (300 million) + *Bifidobacterium breve* strain Yakult (300 million) + galactooligosaccharides (15 g) + EN.	QD	oral & enteral	7	14	21
	Pre-op: standard care alone (ordinary diet). Post-op: standard EN.	QD	enteral	7	14	21
Yokoyama 2016 [68]	Pre-op: Oral *Lactobacillus casei* strain Shirota (40 billion) + *Bifidobacterium breve* strain Yakult (10 billion) + galactooligosaccharides ^a^ (15 g). Post-op: Enteral *Lactobacillus casei* strain Shirota (300 million) + *Bifidobacterium breve* strain Yakult (300 million) + galactooligosaccharides (15 g) + EN.	QD	oral & enteral	7	14	21
	Pre-op: Standard care alone. Post-op: Enteral *Lactobacillus casei* strain Shirota (300 million) + *Bifidobacterium breve* strain Yakult (300 million) + galactooligosaccharides (15 g) + EN.	QD	enteral	NA	14	14
Zhang 2012 [69]	*Bifidobacterium longum* + *Lactobacillus acidophilus* + *Enterococcus faecalis* ^a^ (63 million CFU)	TID	oral	3	NA	3
	Maltodextrin placebo	TID	oral	3	NA	3
Zhao 2017 [70]	*Bifidobacterium* + *Lactobacillus* (6 g) + fiber ^f^ (30 g) + EN	QD	enteral	NA	7	7
	Fiber-free EN control ^b^	QD	enteral	NA	7	7
	Fiber-enriched EN (with 30 g of Shen Jia^TM^ fiber)	QD	enteral	NA	7	7
Zheng 2019 [71]	*Bifidobacterium infantis* (3 million CFU) + *Lactobacillus acidophilus* (3 million CFU) + *Enterococcus faecalis* (3 million CFU) + *Bacillus cereus* (300,000 CFU)	TID	oral	NA	6–7	6–7
	Placebo	TID	oral	NA	6–7	6–7

^a^ This probiotics formulation was studied in more than one RCT (see Figure 2). ^b^ This group was the control arm in the forest plot(s). ^c^ Product brand name is Simbioflora^TM^ (Farmoquimica, Sao Paulo, Brazil). The RCT report lists one of the components as “*L. casei* LPC-37”, but an Internet product search revealed it might be “*L. paracasei* LPC-37”. ^d^ Probiotics were sourced from Atlantic Essential Products (Hauppauge, NY, USA) and prebiotics from Future Ceuticals (Momence, IL, USA). ^e^ Further ingredient details not reported (Inner Mongolia Shuangqi Pharmaceutical Co. Ltd., Inner Mongolia, China). ^f^ Shen Jia^TM^ fiber (ingredient(s) not reported; Beijing Tiantian Yikang Biological Technology Corp. Ltd., Beijing, China). Abbreviations: BID = twice daily, CFU = colony forming units, EN = enteral nutrition, IQR = interquartile range, med. = median, NA = not applicable, NR = not reported, PN = parenteral nutrition, QD = once daily, TID = 3 times daily, Tx = treatment.

**Table 2:** Study and patient characteristics of 36 included RCTs.

Author Year	Sample Size	Country	Study Period	Cancer Types	Funding	Female (%)	Age Mean (Years)	Age Variance
Anderson 2004 [33]	137	UK	NR	Colon (majority) & other GI cancers	NR	48	69	NR
Aso 1992 [34]	48	Japan	1988–1990	Bladder cancer	NR	13	NR	NR
Cho 2019 [35]	36	South Korea	2016–2017	Rectal cancer	public	NR	NR	NR
Consoli 2016 [36]	68	Brazil	2010–2013	Colon cancer	public	55	55	Range 17–83
Diepenhorst 2011 [37]	30	The Netherlands	2005–2006	Periampullary & ampullary pancreatic cancers	NR	50	61	NR
Flesch 2017 [38]	91	Brazil	2013–2015	Colorectal cancer	none	59	63	NR
Franko 2019 [39]	135	USA	2015–2017	Colorectal (70%), hepato-biliary & pancreatic cancers	public	51	62.5	SD 12.1
Horvat 2010 [40]	76	Slovenia	NR	Colorectal cancer	unclear	59	63	Range 29–86
Kanazawa 2005 [41]	54	Japan	2000–2002	Biliary cancers	NR	34	64	NR
Komatsu 2016 [42]	379	Japan	2008–2014	Colorectal cancer	private	42	68	NR
Kotzampassi 2015 [43]	168	Greece	2013–2014	Colorectal cancer	unclear	30	66	NR
Krebs 2016 [45]	60	Slovenia	2009–2012	Colorectal cancer	NR	39	65	Range 43–87
Lages 2018 [46]	40	Brazil	2014–2016	Head & neck cancers	public	19	60.5	NR
Liu 2015 [49]	161	China	2007–2013	Colorectal cancer	public	48	63	NR
Mangell 2012 [50]	72	Sweden	NR	Colon cancer	public	44	72	NR
McNaught 2002 [51]	129	UK	NR	Colorectal (51%) & other GI cancers	NR	42	69	NR
Nomura 2007 [52]	70	Japan	2004–2006	Pancreatic biliary cancers	NR	39	68	Range 30–88
Okazaki 2013 [53]	48	Japan	2009–2011	GI (88%) & hepatobiliary pancreatic cancers	NR	46	79	Range 70–92
Park 2020 [54]	68	South Korea	2016–2018	Sigmoid colon cancer	private	47	61	NR
Polakowski 2019 [55]	73	Brazil	NR	Colorectal cancer	NR	47	60	NR
Rayes 2002 [56]	90	Germany	1997–1999	Hepatic (32%), pancreatic (29%), gastric (24%) & colon cancers	NR	47	61	NR
Rayes 2007 [57]	89	Germany	NR	Pancreatic cancer	NR	44	58.5	NR
Rayes 2012 [58]	19	Germany	2007–2008	Colorectal metastasis (53%), cholangiocellular carcinoma (42%) & liver cancers	NR	26	60	NR
Reddy 2007 [59]	92	UK	NR	Colorectal cancer	private	50	69	NR
Sadahiro 2014 [60]	310	Japan	2008–2011	Colon cancer	private	47	67	NR
Sommacal 2015 [61]	48	Brazil	2010–2012	Periampullary cancers	public	NR	59	Range 44–85
Sugawara 2006 [62]	101	Japan	2003–2005	Biliary cancers	NR	43	63	NR
Tan 2016 [63]	40	Malaysia	2012–2013	Colorectal cancer	private	40	66	NR
Usami 2011 [64]	67	Japan	2005–2008	Hepatic cancer	mixed public & private	10	65	NR
Xu 2019 [65]	60	China	2017–2018	Colorectal cancer	none	37	NR	NR
Yang 2016 [66]	79	China	2011–2012	Colorectal cancer	public	55	63	NR
Yokoyama 2014 [67]	42	Japan	2008–2011	Esophageal cancer	private	12	65.5	Range 25–77
Yokoyama 2016 [68]	45	Japan	2010–2012	Pancreatic & biliary cancers	private	73	65	Range 41–83
Zhang 2012 [69]	60	China	2006–2007	Colorectal cancer	public	60	65	Range 45–87
Zhao 2017 [70]	120	China	2015–2016	Gastric cancer	public	48	66	NR
Zheng 2019 [71]	100	China	2017–2018	Gastric cancer	public	16	62	NR

Abbreviations: GI = gastrointestinal, NR = not reported, SD = standard deviation.

**Table 3:** Quality assessments of the 6 nonrandomized & observational studies.

Author Year	Selection	Comparability	Outcome
Aisu 2015 [72]	★★★		★★
Ding 2018 [73]	★★★★		★★★
Fujio 2020 [74]	★★★★		★★
Mao 2020 [75]	★★★★		★★
Mizuta 2016 [76]	★★★★	★	★★★
Rifatbegovic 2010 [77]	★★★★		★★

NB: More stars indicate higher quality in the Newcastle-Ottawa Scale.

## Data Availability

The data presented in this study are available in detail in the online Supplementary Materials for this article.

## Supplementary Materials

The following are available online at https://www.mdpi.com/article/10.3390/curroncol28060435/s1, Supplementary Materials S1: MEDLINE Search Strategy, Table S1: Interventions/Exposures in the 6 Nonrandomized & Observational Studies, Table S2: Characteristics of the 6 Nonrandomized & Observational Studies, Figure S1: Probiotics formulations studied in more than one RCT, Tables S3: Summary Characteristics of 36 RCTs, Table S4: Additional Results from RCTs, Table S5: Adverse Events and Other Complications in 36 RCTs, Table S6: Results from Nonrandomized & Observational Studies.
