# Supplementary material for: Probiotics Evaluation in Oncological Surgery: A Systematic Review of 36 Randomized Controlled Trials Assessing 21 Diverse Formulations
Source: Curr Oncol. 2021 Dec 7;28(6):5192–214. doi: 10.3390/curroncol28060435 (PMC8700227; doi:10.3390/curroncol28060435)
Supplement: Supplementary file 1 [file curroncol-28-00435-s001.zip › curroncol-1439793-supplementary.pdf]

SUPPLEMENTARY MATERIALS FOR:

Probiotics evaluation in oncological surgery: A systematic review of 36 randomized controlled trials assessing 21 diverse formulations

Cogo E, Elsayed M, Liang V, Cooley K, Guerin C, Psihogios A, Papadogianis P

TABLE OF CONTENTS

MEDLINE Search Strategy 2

Table S1. Interventions/Exposures in the 6 Nonrandomized & Observational Studies 3

Table S2. Characteristics of the 6 Nonrandomized & Observational Studies 5

Figure S1. Probiotics formulations studied in more than one RCT 6

Table S3. Summary Characteristics of 36 RCTs 6

Tables S4. Additional Results from RCTs 9

Table S5. Adverse Events and Other Complications in 36 RCTs 32

Tables S6. Results from Nonrandomized & Observational Studies 40

ABBREVIATIONS:

AE=adverse event, *B.=Bifidobacterium*, BID=twice daily, CI=confidence interval, CFU=colony forming units, EN=enteral nutrition, ERAS=Enhanced Recovery After Surgery, *L.=Lactobacillus*, Lapar.=laparoscopic surgery, NA=not applicable, No.=number, NR=not reported, OC=other complication, post-op=post-operative, PN=parenteral nutrition, pre-op=pre-operative, QD=once daily, RCT=randomized controlled trial, SD=standard deviation, SE=standard error, TID=three times daily, tx=treatment, var=variance.

MEDLINE Search Strategy

(Ovid; 1946-present)

1. fish oils/ or fatty acids, omega-3/ or docosahexaenoic acids/ or eicosapentaenoic acid/
2. arginine/
3. glutamine/
4. RNA/
5. exp Mistletoe/ or Viscum album/
6. vitamin k/ or vitamin k 1/ or vitamin k 2/ or vitamin k 3/
7. probiotics/ or synbiotics/ or exp Lactobacillus/ or exp Bifidobacterium/ or Saccharomyces boulardii/
8. amino acids, branched-chain/ or isoleucine/ or leucine/ or valine/
9. Lycopersicon esculentum/
10. Ubiquinone/
11. ((fish adj oil*) or (omega adj 3*) or docosahexaenoic or eicosapentaenoic or immunonutrition or arginine or glutamine or mistletoe or Viscum album or (vitamin adj k*) or probiotic* or synbiotic* or Lactobacill* or Bifidobacteri* or Saccharomyces boulardii or branched chain amino acid* or leucine or isoleucine or valine or tomato or lycopene or coq10 or (coenzyme adj q10) or ubiquino*).ti,ab,kf.
12. or/1-11
13. (cancer* or carcinoma* or tumor* or tumour* or neoplas* or adenoma* or metastas* or malignan* or oncolog*).mp.
14. exp Neoplasms/
15. exp Precancerous Conditions/
16. 14 not 15
17. ((anti adj cancer) or anticancer). ti,ab,kf.
18. ((anti adj neoplastic) or antineoplastic). ti,ab,kf.
19. exp Antineoplastic Agents/
20. 13 or 16 or 17 or 18 or 19
21. 12 and 20
22. limit 12 to cancer
23. 21 or 22
24. exp perioperative care/ or exp perioperative period/ or exp Specialties, Surgical/ or exp Surgical Procedures, Operative/ or exp Postoperative Complications/
25. (surgery or surgical or surgeries or resect* or perioperative* or preoperative* or operative* or postoperative*). ti,ab,kf.
26. 24 or 25
27. 23 and 26
28. exp Animals/ not (exp Animals/ and Humans/)
29. 27 not 28
30. limit 29 to english language
31. (randomized controlled trial or controlled clinical trial).pt.
32. (randomized or randomly or placebo).ab.
33. (trial or groups).ab.
34. drug therapy.fs.
35. 31 or 32 or 33 or 34
36. 30 and 35
37. exp case-control studies/ or exp cohort studies/
38. longitudinal studies/ or follow-up studies/ or prospective studies/ or retrospective studies/ or Control Groups/ or Matched-Pair Analysis/ or Comparative studies/
39. 37 or 38
40. 30 and 39
41. limit 30 to (clinical trial, phase ii or clinical trial, phase iii or clinical trial, phase iv or comparative study or controlled clinical trial or observational study or randomized controlled trial)
42. 36 or 40 or 41

Table S1. Interventions/Exposures in the 6 Nonrandomized & Observational Studies

Author Year	Interventions/Exposures* and Comparators	Freq. of dose	Route of admin.	Tx duration Pre-op	Tx duration Post-op	Tx duration TOTAL (days)
Aisu 2015	*Enterococcus faecalis* T110 (12 mg) + *Clostridium butyricum* TO-A (60 mg) + *Bacillus mesentericus* TO-A (60 mg) (BIO-THREE)	QD	oral	3-15	yes (duration NR)	NR
	No added tx	NA	NA	NA	NA	NA
Ding 2018	*Clostridium butyricum* MIYAIRI (40 mg) + ERAS multidisciplinary periop program	TID	oral	3	7	10
	ERAS multidisciplinary periop program (without probiotics)	NA	NA	NA	NA	NA
Fujio 2020	ERAS multidisciplinary, 17-component periop protocol. Synbiotic component: *L. casei* Shirota (Yakult) + *Bacillus mesentericus* + *Clostridium butyricum* + *Enterococcus faecalis* (BIO-THREE) + oligosaccharides + dietary fiber + glutamine + antiflatulent drug	NR	oral	3	NA	3
	Pre-ERAS periop program	NA	NA	NA	NA	NA
Mao 2020	*Bifidobacteria*	NR	NR	yes (duration NR)	NA	NR
	No added tx	NA	NA	NA	NA	NA
Mizuta 2016	*Bifidobacterium longum* BB536 (5×10^{10} CFU)	QD	oral	7-14	14	21-28

	No added tx	NA	NA	NA	NA	NA
Rifatbegovic 2010	*Lactobacillus plantarum* 2362 + *L. paracasei subsp paracase* 19 + *Pediacoccus pentoseceus* 5-33:3 + *Leuconostoc mesenteroides* 32-77:1 + *L. raffinolactis*	NR	oral	3	7	10
	No added tx	NA	NA	NA	NA	NA

*Dosages are presented when they were reported in the studies.
Abbreviations: CFU=colony forming units, NA=not applicable, NR= not reported, QD=once daily, TID=3 times daily, Tx=treatment.

Table S2. Characteristics of the 6 Nonrandomized & Observational Studies

Author Year	Country of conduct	Study design	Funding	Study period	Cancer type	Sample size	Age (years; mean)	Age variance	Female (%)
Aisu 2015	Japan	non-randomized controlled trial	private	2009-2013	Colorectal cancer	156	69	NR	42
Ding 2018[a]	China	cohort	public	2017	Liver cancer	Open: 20<br>Lapar.: 49	57	NR	38
Fujio 2020	Japan	cohort	NR	2011-2014	Hepatocellular carcinoma	97	67 (median)	Range 40-86	17
Mao 2020	China	cohort	unclear	2014-2019	Colorectal cancer	122	59 (median)	Range 28-82	NR
Mizuta 2016	Japan	non-randomized controlled trial[b]	NR	2008-2012	Colorectal cancer	45	70	NR	51
Rifatbegovic 2010	Bosnia and Herzegovina; France; Slovenia	non-randomized controlled trial	NR	2006-2008	Hepatocellular carcinoma	120	NR	NR	NR

[a]Extracted as 2 cohorts, based on type of hepatectomy. Extracted the report's 4 ERAS groups (not their "Control" groups pre-ERAS).
[b]Study was re-classified as nonrandomized here since the chemotherapy/radiation patients were not eligible/included in the review.
Abbreviations: ERAS=Enhanced Recovery After Surgery, Lapar.=laparoscopic surgery, NR=not reported.

Figure S1. Probiotics formulations studied in more than one RCT

6 Interventions Were Studied in Multiple RCTs	
**In 7 RCTs:**<br>- *Lactobacillus casei* strain Shirota,<br>- *Bifidobacterium breve* strain Yakult, and<br>- galactooligosaccharides combination.	**In 3 RCTs:**<br>- *Lactobacillus acidophilus* NCFM,<br>- *Lactobacillus rhamnosus* HN001,<br>- *Lactobacillus paracasei* LPC-37,<br>- *Bifidobacterium lactis* HN019, and<br>- fructooligosaccharides combination.
**In 4 RCTs:**<br>- *Pediacoccus pentosaceus* 5-33:3,<br>- *Leuconostoc mesenteroides* 77:1,<br>- *Lactobacillus paracasei* subsp. *paracasei* F19,<br>- *Lactobacillus plantarum* 2362,<br>- betaglucan,<br>- inulin,<br>- pectin, and<br>- resistant starch combination.	**In 2 RCTs:**<br>- *Lactobacillus acidophilus* La5,<br>- *Bifidobacterium lactis* Bb-12,<br>- *Streptococcus thermophilus,*<br>- *Lactobacillus bulgaricus,* and<br>- oligofructose combination.
**In 3 RCTs:**<br>- *Lactobacillus plantarum* 299v, and<br>- oatmeal combination.	**In 2 RCTs:**<br>- *Bifidobacterium longum,*<br>- *Lactobacillus acidophilus,* and<br>- *Enterococcus faecalis* combination.

Table S3. Summary Characteristics of 36 RCTs

Characteristic		No.	%
**CANCER TYPE**			
	Colorectal	19	53
	Hepato-biliary or Pancreatic	8	22
	Gastric or Esophageal	3	8
	Combines multiple Digestive System types	4	11
	Head and neck	1	3
	Bladder	1	3

**SURGICAL PERIOD OF USE**			
	Pre-operative only	9	25
	Post-operative only	7	19
	Mixed periods	20	56
**ROUTE OF ADMINISTRATION**			
	Oral	30	83
	Enteral (excluding oral)	6	17
**PROBIOTICS FORMULATION**			
	Single probiotic bacteria	7	19
	Multi-strain combination	29	81
**AGE (mean/median)**			
	55-64 years	17	47
	65-79 years	16	44
	Not reported	3	8
**FEMALE**			
	10-29 %	6	17
	30-49 %	19	53
	50-73 %	9	25
	Not reported	2	6
**COUNTRY**			
	Japan	10	28
	China	6	17
	Brazil	5	14

	Germany	3	8
	UK	3	8
	Slovenia	2	6
	South Korea	2	6
	Greece	1	3
	Malaysia	1	3
	Netherlands	1	3
	Sweden	1	3
	USA	1	3
**SAMPLE SIZE**			
	19-49	10	28
	50-99	16	44
	100-199	8	22
	200-379	2	6
**FUNDING**			
	Public	11	31
	Private or Mixed	8	22
	None	2	6
	Unclear	2	6
	Not reported	13	36
**YEAR OF PUBLICATION**			
	2001-2010<br>(plus 1 RCT from 1992)	9	25
	2011-2020	26	72

Tables S4. Additional Results from RCTs

Alphabetical List of Included RCTs

NB: The number below corresponds to the reference number cited in the main report's References section:

33. Anderson AD, McNaught CE, Jain PK, MacFie J. Randomised clinical trial of synbiotic therapy in elective surgical patients. *Gut.* 2004;53(2):241-245.
34. Aso Y, Akazan H. Prophylactic effect of a Lactobacillus casei preparation on the recurrence of superficial bladder cancer. BLP Study Group. *Urol Int.* 1992;49(3):125-129.
35. Cho JR, Yoon BJ, Oh HK. Effect of Probiotics on Bowel Function Restoration After Ileostomy Reversal in Patients with Rectal Cancer: A Double-Blind Randomized Controlled Trial. *Gastroenterology.* 2019;156(6 S1):S-1421-S-1421.
36. Consoli ML, da Silva RS, Nicoli JR, et al. Randomized Clinical Trial: Impact of Oral Administration of Saccharomyces boulardii on Gene Expression of Intestinal Cytokines in Patients Undergoing Colon Resection. *Jpen: Journal of Parenteral & Enteral Nutrition.* 2016;40(8):1114-1121.
37. Diepenhorst GM, van RO, Besselink MG, et al. Influence of prophylactic probiotics and selective decontamination on bacterial translocation in patients undergoing pancreatic surgery: a randomized controlled trial. *Shock.* 2011;35(1):9-16.
38. Flesch AT, Tonial ST, Contu PC, et al. Perioperative synbiotics administration decreases postoperative infections in patients with colorectal cancer: a randomized, double-blind clinical trial. *Revista do Colegio Brasileiro de Cirurgioes.* 2017;44(6):567-573.
39. Franko J, Raman S, Krishnan N, et al. Randomized Trial of Perioperative Probiotics Among Patients Undergoing Major Abdominal Operation. *J Am CollSurg.* 2019;229(6):533-540.
40. Horvat M, Krebs B, Potrč S, et al. Preoperative synbiotic bowel conditioning for elective colorectal surgery. *Wiener Klinische Wochenschrift.* 2010;122 Suppl 2:26-30.
41. Kanazawa H, Nagino M, Kamiya S, et al. Synbiotics reduce postoperative infectious complications: a randomized controlled trial in biliary cancer patients undergoing hepatectomy. *Langenbecks Archives of Surgery.* 2005;390(2):104-113.
42. Komatsu S, Sakamoto, Komatsu S, et al. Efficacy of perioperative synbiotics treatment for the prevention of surgical site infection after laparoscopic colorectal surgery: a randomized controlled trial. *Surgery Today.* 2016;46(4):479-490.
43. Kotzampassi K, Stavrou G, Damoraki G, et al. A Four-Probiotics Regimen Reduces Postoperative Complications After Colorectal Surgery: A Randomized, Double-Blind, Placebo-Controlled Study. *World Journal of Surgery.* 2015;39(11):2776-2783.
44. Krebs B, Horvat M, Golle A, et al. A randomized clinical trial of synbiotic treatment before colorectal cancer surgery. *American Surgeon.* 2013;79(12):E340-E342.
45. Krebs B. Prebiotic and Synbiotic Treatment before Colorectal Surgery--Randomised Double Blind Trial. *Collegium Antropologicum.* 2016;40(1):35-40.
46. Lages PC, Generoso SV, Correia M, et al. Postoperative symbiotic in patients with head and neck cancer: a double-blind randomised trial. *British Journal of Nutrition.* 2018;119(2):190-195.
47. Liu Z, Qin H, Yang Z, et al. Randomised clinical trial: the effects of perioperative probiotic treatment on barrier function and post-operative infectious complications in colorectal cancer surgery - a double-blind study. *Aliment Pharmacol Ther.* 2011;33(1):50-63.
48. Liu ZH, Huang MJ, Zhang XW, et al. The effects of perioperative probiotic treatment on serum zonulin concentration and subsequent postoperative infectious complications after colorectal cancer surgery: a double-center and double-blind randomized clinical trial. *American Journal of Clinical Nutrition.* 2013;97(1):117-126.
49. Liu Z, Li C, Huang M, et al. Positive regulatory effects of perioperative probiotic treatment on postoperative liver complications after colorectal liver metastases surgery: a double-center and double-blind randomized clinical trial. *BMC Gastroenterology.* 2015;15:34.
50. Mangell P, Thorlacius H, Syk I, et al. Lactobacillus plantarum 299v does not reduce enteric bacteria or bacterial translocation in patients undergoing colon resection. *Digestive Diseases & Sciences.* 2012;57(7):1915-1924.
51. McNaught CE, Woodcock NP, Macfie J, Mitchell CJ. A prospective randomised study of the probiotic Lactobacillus plantarum 299V on indices of gut barrier function in elective surgical patients. *Gut.* 2002;51(6):827-831.
52. Nomura T, Tsuchiya Y, Nashimoto A, et al. Probiotics reduce infectious complications after pancreaticoduodenectomy. *Hepato-Gastroenterology.* 2007;54(75):661-663.
53. Okazaki M, Matsukuma S, Suto R, et al. Perioperative synbiotic therapy in elderly patients undergoing gastroenterological surgery: a prospective, randomized control trial. *Nutrition.*

2013;29(10):1224-1230.
54. Park IJ, Lee JH, Kye BH, et al. Effects of PrObiotics on the Symptoms and Surgical ouTComes after Anterior REsection of Colon Cancer (POSTCARE): A Randomized, Double-Blind, Placebo-Controlled Trial. *J Clin Med.* 2020;9(7).
55. Polakowski CB, Kato M, Preti VB, et al. Impact of the preoperative use of synbiotics in colorectal cancer patients: A prospective, randomized, double-blind, placebo-controlled study. *Nutrition.* 2019;58:40-46.
56. Rayes N, Hansen S, Seehofer D, et al. Early enteral supply of fiber and Lactobacilli versus conventional nutrition: a controlled trial in patients with major abdominal surgery. *Nutrition.* 2002;18(7-8):609-615.
57. Rayes N, Seehofer D, Theruvath T, et al. Effect of enteral nutrition and synbiotics on bacterial infection rates after pylorus-preserving pancreatoduodenectomy: a randomized, double-blind trial. *Ann Surg.* 2007;246(1):36-41.
58. Rayes N, Pilarski T, Stockmann M, et al. Effect of pre- and probiotics on liver regeneration after resection: a randomised, double-blind pilot study. *BenefMicrobes.* 2012;3(3):237-244.
59. Reddy BS, Macfie J, Gatt M, et al. Randomized clinical trial of effect of synbiotics, neomycin and mechanical bowel preparation on intestinal barrier function in patients undergoing colectomy. *Br J Surg.* 2007;94(5):546-554.
60. Sadahiro S, Suzuki, Sadahiro S, et al. Comparison between oral antibiotics and probiotics as bowel preparation for elective colon cancer surgery to prevent infection: prospective randomized trial. *Surgery.* 2014;155(3):493-503.
61. Sommacal HM, Bersch VP, Vitola SP, et al. Perioperative synbiotics decrease postoperative complications in periampullary neoplasms: a randomized, double-blind clinical trial. *Nutrition & Cancer.* 2015;67(3):457-462.
62. Sugawara G, Nagino M, Nishio H, et al. Perioperative synbiotic treatment to prevent postoperative infectious complications in biliary cancer surgery: a randomized controlled trial. *Ann Surg.* 2006;244(5):706-714.
63. Tan CK, Said S, Rajandram, et al. Pre-surgical Administration of Microbial Cell Preparation in Colorectal Cancer Patients: A Randomized Controlled Trial. *World Journal of Surgery.* 2016;40(8):1985-1992.
64. Usami M, Miyoshi M, Kanbara Y, et al. Effects of perioperative synbiotic treatment on infectious complications, intestinal integrity, and fecal flora and organic acids in hepatic surgery with or without cirrhosis. *Jpen: Journal of Parenteral & Enteral Nutrition.* 2011;35(3):317-328.
65. Xu Q, Xu P, Cen Y, Li W. Effects of preoperative oral administration of glucose solution combined with postoperative probiotics on inflammation and intestinal barrier function in patients after colorectal cancer surgery. *Oncol Lett.* 2019;18(1):694-698.
66. Yang Y, Xia Y, Chen H, et al. The effect of perioperative probiotics treatment for colorectal cancer: short-term outcomes of a randomized controlled trial. *Oncotarget.* 2016;7(7):8432-8440.
67. Yokoyama Y, Nishigaki E, Abe T, et al. Randomized clinical trial of the effect of perioperative synbiotics versus no synbiotics on bacterial translocation after oesophagectomy. *British Journal of Surgery.* 2014;101(3):189-199.
68. Yokoyama Y, Miyake T, Kokuryo T, et al. Effect of Perioperative Synbiotic Treatment on Bacterial Translocation and Postoperative Infectious Complications after Pancreatoduodenectomy. *Digestive Surgery.* 2016;33(3):220-229.
69. Zhang J-W, Du P, Gao J, et al. Preoperative probiotics decrease postoperative infectious complications of colorectal cancer. *American Journal of the Medical Sciences.* 2012;343(3):199-205.
70. Zhao R, Wang Y, Huang Y, et al. Effects of fiber and probiotics on diarrhea associated with enteral nutrition in gastric cancer patients: A prospective randomized and controlled trial. *Medicine.* 2017;96(43):e8418.
71. Zheng C, Chen T, Wang Y, et al. A randomised trial of probiotics to reduce severity of physiological and microbial disorders induced by partial gastrectomy for patients with gastric cancer. *J Cancer.* 2019;10(3):568-576.

Table for Bacteremia (N=12 RCTs)

Author Year	Interventions & Comparators	Sample size	Number of events	Follow-up time (days)	Name of outcome	p-value[a]
Kanazawa 2005	*Lactobacillus casei* strain Shirota + *Bifidobacterium breve* strain Yakult + galactooligosaccharides + EN + PN	21	1	30	Bacteremia	NR
	Standard EN + PN	23	4	30	Bacteremia	
Kotzampassi 2015	*Lactobacillus acidophilus* LA-5 + *L. plantarum* + *Bifidobacterium lactis* BB-12 + *Saccharomyces boulardii*	84	6	30	Bacteremia	p=0.583
	Glucose polymer placebo	80	8	30	Bacteremia	
Lages 2018	*Lactobacillus paracasei* LPC-31 + *L. rhamnosus* HN00l + *L. acidophilus* NCFM + *Bifidobacterium lactis* HN019 + fructooligosaccharides + standard EN	18	0	30	Bacteremia	p>0.05
	Maltodextrin placebo + standard EN	18	1	30	Bacteremia	
Liu 2015	*Lactobacillus plantarum* CGMCC No. 1258 + *L. acidophilus* LA-11 + *Bifidobacterium longum* BL-88	66	7	30	Central line infections	p= 0.777
	Maltodextrin placebo	68	6	30	Central line infections	
Okazaki 2013	*Lactobacillus casei* strain Shirota + *Bifidobacterium breve* strain Yakult + galactooligosaccharides	25	0	30	Bacteremia	NR
	Standard care alone	23	1	30	Bacteremia	
Rayes 2002	Live *Lactobacillus plantarum* 299 + oat fiber + EN	30	0	45	Sepsis	NR
	Heat-killed *Lactobacillus plantarum* 299 + oat fiber + EN	30	1	45	Sepsis	
	Standard total parenteral nutrition or fiber-free EN control	30	1	45	Sepsis	
Rayes 2007	*Pediacoccus pentosaceus* 5-33:3 + *Leuconostoc mesenteroides* 32–77:1 + *Lactobacillus paracasei* subsp. *paracasei* 19 + *L. plantarum* 2362 + betaglucan + inulin + pectin + resistant starch fibers + EN	40	0	30	Sepsis	NR
	Betaglucan + inulin + pectin + resistant starch fibers + EN	40	2	30	Sepsis	
Sommacal 2015	*Lactobacillus acidophilus* 10 + *L. rhamnosus* HS 111 + *L. casei* 10 + *Bifidobacterium bifidum* + fructooligosaccharides	23	0	50	Septic shock	NR
	Sucrose placebo	23	3	50	Septic shock	
Tan 2016	*Lactobacillus acidophilus* BCMC12130 + *L. casei* BCMC12313 + *L.*	20	1	30	Line infections	p=1.00

	lactis BCMC12451 + *Bifidobacterium bifidum* BCMC02290 + *B. longum* BCMC02120 + *B. infantis* BCMC02129					
	Placebo	20	1	30	Line infections	
Yang 2016	*Bifidobacterium longum* + *Lactobacillus acidophilus* + *Enterococcus faecalis*	30	3	25	Bacteremia	p= 0.053
	Maltodextrin + sucrose placebo	30	9	25	Bacteremia	
Yokoyama 2014	Pre-op: oral or enteral *Lactobacillus casei* strain Shirota + *Bifidobacterium breve* strain Yakult + galactooligosaccharides.<br><br>Post-op: enteral *Lactobacillus casei* strain Shirota + *Bifidobacterium breve* strain Yakult + galactooligosaccharides + EN.	21	2	30	Bacteremia	p=0.147
	Pre-op: standard care alone (ordinary diet).<br><br>Post-op: standard EN.	21	0	30	Bacteremia	
Zhang 2012	*Bifidobacterium longum* + *Lactobacillus acidophilus* + *Enterococcus faecalis*	30	2	9	Bacteremia	p=0.02
	Maltodextrin placebo	30	9	9	Bacteremia	

[a]p-value between groups.

Table for Anastomotic Leakage or Abdominal Abscess (N=16 RCTs)

Author Year	Interventions & Comparators	Sample size	Number of events	Name of outcome
Consoli 2016	*Saccharomyces boulardii*	15	0	Intra-abdominal abscess
	Standard care alone	18	4	Intra-abdominal abscess
Diepenhorst 2011	*Bifidobacterium bifidum* + *B. infantis* + *Lactobacillus acidophilus* + *L. casei* + *L. salivarius* + *L. lactis*	10	0	Intra-abdominal abscess
	Standard tx control	10	0	Intra-abdominal abscess
Flesch 2017	*Lactobacillus acidophilus* NCFM + *L. rhamnosus* HN001 + *L. paracasei* LPC-37 + *Bifidobacterium lactis* HN019 + fructooligosaccharides	49	0	Intra-abdominal abscess
	Maltodextrin placebo	42	3	Intra-abdominal abscess
Kanazawa 2005	*Lactobacillus casei* strain Shirota + *Bifidobacterium breve* strain Yakult + galactooligosaccharides + EN + PN	21	2	Intra-abdominal abscess
	Standard EN + PN	23	4	Intra-abdominal abscess
Komatsu 2016	*Lactobacillus casei* strain Shirota + *Bifidobacterium breve* strain Yakult + galactooligosaccharides	168	18	30- days Organ/space infection
	Standard care alone	194	21	30- days Organ/space infection
	Lactobacillus casei strain Shirota + *Bifidobacterium breve* strain Yakult + galactooligosaccharides	168	12	30-day surgical site infection with leakage
	Standard care alone	194	12	30-day surgical site infection with leakage
Mangell 2012	*Lactobacillus plantarum* 299v in an oatmeal-based drink	32	0	Intra-abdominal abscess (severe)
	Oatmeal-based placebo drink without probiotics	32	1	Intra-abdominal abscess (severe)
Okazaki 2013	*Lactobacillus casei* strain Shirota + *Bifidobacterium breve* strain Yakult + galactooligosaccharides	25	1	Organ/space infection
	Standard care alone	23	3	Organ/space infection
Rayes 2002	Live *Lactobacillus plantarum* 299 + oat fiber + EN	30	0	Peritonitis
	Standard total parenteral nutrition or fiber-free EN control	30	1	Peritonitis

Rayes 2007	*Pediacoccus pentosaceus* 5-33:3 + *Leuconostoc mesenteroides* 32–77:1 + *Lactobacillus paracasei* subsp. *paracasei* 19 + *L. plantarum* 2362 + betaglucan + inulin + pectin + resistant starch fibers + EN	40	0	Peritonitis
	Betaglucan + inulin + pectin + resistant starch fibers + EN	40	5	Peritonitis
Rayes 2012	*Pediacoccus pentosaceus* 5-33:3 + *Leuconostoc mesenteroides* 32–77:1 + *Lactobacillus paracasei* subsp. *paracasei* 19 + *L. plantarum* 2362 + betaglucan + inulin + pectin + resistant starch fibers + EN	9	1	hepatic abscess alone infection
	Betaglucan + inulin + pectin + resistant starch fibers + EN	10	1	hepatic abscess alone infection
Reddy 2007	*Lactobacillus acidophilus* La5 + *L. bulgaricus* + *Bifidobacterium lactis* Bb-12 + *Streptococcus thermophilus* + oligofructose + neomycin + mechanical bowel preparation	20	0	intra-abdominal collection
	Neomycin + mechanical bowel preparation control	22	1	intra-abdominal collection
Sadahiro 2014	*Bifidobacterium bifidum* + maltooligosaccharide [plus single IV dose of flomoxef; & standard mechanical bowel preparation]	100	1	Leakage with abscess
	Standard care alone control [plus single IV dose of flomoxef; & standard mechanical bowel preparation. No probiotic or oral antibiotics]	95	2	Leakage with abscess
	Bifidobacterium bifidum + maltooligosaccharide [plus single IV dose of flomoxef; & standard mechanical bowel preparation]	100	4	Organ/space surgical site infection
	Standard care alone control [plus single IV dose of flomoxef; & standard mechanical bowel preparation. No probiotic or oral antibiotics]	95	5	Organ/space surgical site infection
Usami 2011	*Lactobacillus casei* strain Shirota + *Bifidobacterium breve* strain Yakult + galactooligosaccharides [+ PN for 4 days post-op]	32	0	Intra-abdominal abscess
	Standard care alone [+ PN for 4 days post-op]	29	2	Intra-abdominal abscess
Yang 2016	*Bifidobacterium longum* + *Lactobacillus acidophilus* + *Enterococcus faecalis*	30	1	anastomotic leakage
	Maltodextrin + sucrose placebo	30	1	anastomotic leakage
Yokoyama 2014	Pre-op: oral or enteral *Lactobacillus casei* strain Shirota + *Bifidobacterium breve* strain Yakult + galactooligosaccharides.<br><br>Post-op: enteral *Lactobacillus casei* strain Shirota + *Bifidobacterium breve* strain Yakult + galactooligosaccharides + EN.	21	3	Anastomotic leakage
	Pre-op: standard care alone (ordinary diet).	21	2	Anastomotic leakage

	Post-op: standard EN.			
	Pre-op: oral or enteral *Lactobacillus casei* strain Shirota + *Bifidobacterium breve* strain Yakult + galactooligosaccharides.<br><br>Post-op: enteral *Lactobacillus casei* strain Shirota + *Bifidobacterium breve* strain Yakult + galactooligosaccharides + EN.	21	2	Mediastinal or abdominal abscess
	Pre-op: standard care alone (ordinary diet).<br><br>Post-op: standard EN.	21	0	Mediastinal or abdominal abscess
Zhang 2012	*Bifidobacterium longum* + *Lactobacillus acidophilus* + *Enterococcus faecalis*	30	0	Anastomotic leakage
	Maltodextrin placebo	30	2	Anastomotic leakage
	Bifidobacterium longum + *Lactobacillus acidophilus* + *Enterococcus faecalis*	30	2	Intra-abdominal abscesses
	Maltodextrin placebo	30	1	Intra-abdominal abscesses

Table for WBCs (N=12 RCTs)

Author Year	Interventions & Comparators	Sample size	Preop value (mean)	Var. type	Var. – Preop	Follow-up value (mean)	Var. – Follow-up	Unit	Follow-up time (days)	p-value[a]
Lages 2018	*Lactobacillus paracasei* LPC-31 + *L. rhamnosus* HN00l + *L. acidophilus* NCFM + *Bifidobacterium lactis* HN019 + fructooligosaccharides + standard EN	18	8.4	SD	2.9	11.2	3.1	10^9/l	5-7	p>0.05
	Maltodextrin placebo + standard EN	18	8.7	SD	3	11.5	5	10^9/l	5-7	
Yoko-yama 2016	Pre-op: Oral *Lactobacillus casei* strain Shirota + *Bifidobacterium breve* strain Yakult + galactooligosaccharides. Post-op: Enteral *Lactobacillus casei* strain Shirota + *Bifidobacterium breve* strain Yakult + galactooligosaccharides + EN.	22	5.1	range	2.8–8.9	9.2	4.9–17.3	10^3µl	3	p=0.92
	Pre-op: Standard care alone. Post-op: Enteral *Lactobacillus casei* strain Shirota + *Bifidobacterium breve* strain Yakult + galactooligosaccharides + EN.	22	5.4	range	2.9–7.5	8.5	3.7–18.0	10^3µl	3	
Yang 2016	*Bifidobacterium longum* + *Lactobacillus acidophilus* + *Enterococcus faecalis*	30	6.14	SD	1.47	1.64	1.78	10^9/L	12	p=0.37
	Maltodextrin + sucrose placebo	30	6.56	SD	2.54	2.11	2.26	10^9/L	12	
Yoko-yama 2014	Pre-op: oral or enteral *Lactobacillus casei* strain Shirota + *Bifidobacterium breve* strain Yakult + galactooligosaccharides.<br><br>Post-op: enteral *Lactobacillus casei* strain Shirota + *Bifidobacterium breve* strain Yakult + galactooligosaccharides	21	5000	NR	NR	9500	NR	per µl	8	p>0.05

	+ EN.									
	Pre-op: standard care alone (ordinary diet).<br><br>Post-op: standard EN.	21	6500	NR	NR	8800	NR	per µl	8	
Usami 2011	*Lactobacillus casei* strain Shirota + *Bifidobacterium breve* strain Yakult + galactooligosaccharides [+ PN for 4 days post-op]	32	47.2	SD	13.4	61.3	16.8	10^2/µL	13-15	p>0.05
	Standard care alone [+ PN for 4 days post-op]	29	57.3	SD	15.9	66.2	18.6	10^2/µL	13-15	
Horvat 2010	*Pediacoccus pentosaceus* 5-33:3 + *Leuconostoc mesenteroides* 32–77:1 + *Lactobacillus paracasei* subsp. *paracasei* 19 + *L. plantarum* 2362 + betaglucan + inulin + pectin + resistant starch fibers [without mechanical bowel preparation]	20	7.3	range	4.4-13.1	9.7	5.1–17.5	10^9/l	5	p=0.53
	Mechanical bowel preparation control	20	5.9	range	3.8-10.8	8.3	5.0–12.6	10^9/l	5	
	Heat-inactivated lactobacilli + betaglucan + inulin + pectin + resistant starch fibers [without mechanical bowel preparation]	28	7.1	range	3.9-11.9	10.6	6.0–55.5	10^9/l	5	
Kana-zawa 2005	*Lactobacillus casei* strain Shirota + *Bifidobacterium breve* strain Yakult + galactooligosaccharides + EN + PN	21	6.1	SD	2.4	7.3	2.3	10^3/µl	14	p>0.05
	Standard EN + PN	23	5.6	SD	2	8	3	10^3/µl	14	
Suga-wara 2006	Pre-op: Oral *Lactobacillus casei* strain Shirota + *Bifidobacterium breve* strain Yakult + galactooligosaccharides.<br>Post-op: Enteral *Lactobacillus casei* strain Shirota + *Bifidobacterium breve* strain Yakult + galactooligosaccharides + standard EN + PN.	41	6600	NR	NR	5900	NR	per µl	14	p>0.05
	Pre-op: Standard care alone.	40	6800	NR	NR	6100	NR	per µl	14	

	Post-op: Enteral *Lactobacillus casei* strain Shirota + *Bifidobacterium breve* strain Yakult + galactooligosaccharides + standard EN + PN.									
Park 2020	*Bifidobacterium animalis* subsp. *lactis* HY8002 + *Lactobacillus casei* HY2782 + *L. plantarum* HY7712 + xylooligosaccharides + fructooligosaccharides	29	6.14	SD	1.4	5.76	1.39	NR	28	p=0.57
	Xylooligosaccharides + fructooligosaccharides	31	6.97	SD	2.17	6.82	2.3	NR	28	
Zheng 2019	*Bifidobacterium infantis* + *Lactobacillus acidophilus* + *Enterococcus faecalis* + *Bacillus cereus*	50	NA	NA	NA	46[b]	NA	NA	7	NR
	Placebo	50	NA	NA	NA	41[b]	NA	NA	7	
Xu 2019	Bifidus-triple viable preparation + glucose solution	30	4.3	SD	0.5	5.6	0.9	10^9/l	8	p<0.05
	Glucose solution	30	4.2	SD	0.5	7.1	1.3	10^9/l	8	
Rayes 2002	Live *Lactobacillus plantarum* 299 + oat fiber + EN	30	7.8	SE	0.8	10.3	0.8	per µL	12	p>0.05
	Standard total parenteral nutrition or fiber-free EN control	30	7.1	SE	0.5	9.8	0.9	per µL	12	
	Heat-killed *Lactobacillus plantarum* 299 + oat fiber + EN	30	7.7	SE	0.7	10.6	0.8	per µL	12	

[a]p-value between groups.

[b]Number of participants restoring to normal range for leukocytes (3.5-9.5 × 10^9 cells/L).

Table for Lymphocytes (N=6 RCTs)

Author Year	Interventions & Comparators	Sample size	Preop value (mean)	Var. type	Var. – Preop	Follow-up value (mean)	Var. – Follow-up	Unit	Follow-up time (days)	p-value[a]
Horvat 2010	*Pediacoccus pentosaceus* 5-33:3 + *Leuconostoc mesenteroides* 32–77:1 + *Lactobacillus paracasei* subsp. *paracasei* 19 + *L. plantarum* 2362 + betaglucan + inulin + pectin + resistant starch fibers [without mechanical bowel preparation]	20	0.45[b]	range	0.17-1.00	0.15[b]	0.07–0.34	NA	5	p=0.82
	Heat-inactivated lactobacilli + betaglucan + inulin + pectin + resistant starch fibers [without mechanical bowel preparation]	28	0.32[b]	range	0.14-0.62	0.17[b]	0.05–0.30	NA	5	
	Mechanical bowel preparation control	20	0.44[b]	range	0.09–0.87	0.18[b]	0.07–0.32	NA	5	
Park 2020	*Bifidobacterium animalis* subsp. *lactis* HY8002 + *Lactobacillus casei* HY2782 + *L. plantarum* HY7712 + xylooligosaccharides + fructooligosaccharides	29	32.31	SD	7.18	34.91	7.88	NR	28	p>0.05
	Xylooligosaccharides + fructooligosaccharides	31	31.69	SD	9.73	31.78	7.05	NR	28	
Rayes 2002	Live *Lactobacillus plantarum* 299 + oat fiber + EN	30	1,280	SE	114	996	103	per µL	12	p<0.05
	Heat-killed *Lactobacillus plantarum* 299 + oat fiber + EN	30	1079	SE	169	956	95	per µL	12	
	Standard total parenteral nutrition or	30	1131	SE	117	1284	175	per µL	12	

	fiber-free EN control									
Sugawara 2006	Pre-op: Oral *Lactobacillus casei* strain Shirota + *Bifidobacterium breve* strain Yakult + galactooligosaccharides. Post-op: Enteral *Lactobacillus casei* strain Shirota + *Bifidobacterium breve* strain Yakult + galactooligosaccharides + standard EN + PN.	41	1404	SD	226	1721	285	per µl	14	NR
	Pre-op: Standard care alone. Post-op: Enteral *Lactobacillus casei* strain Shirota + *Bifidobacterium breve* strain Yakult + galactooligosaccharides + standard EN + PN.	40	1550	NR	NR	1550	NR	per µl	14	
Zhao 2017	*Bifidobacterium* + *Lactobacillus* + fiber + EN	40	1.13	SD	0.32	0.96	0.16	10^9/L	7	p=0.58
	Fiber-enriched EN	40	1.15	SD	0.34	0.94	0.16	10^9/L	7	
	Fiber-free EN control	40	1.25	SD	0.42	1.01	0.28	10^9/L	7	
Zheng 2019	*Bifidobacterium infantis* + *Lactobacillus acidophilus* + *Enterococcus faecalis* + *Bacillus cereus*	50	NA	NA	NA	7[c]	NA	NA	7	NR
	Placebo	50	NA	NA	NA	1[c]	NA	NA	7	

[a]p-value between groups.

[b]Lymphocyte-to-granulocyte ratio.

[c]Number of participants restoring to normal range for lymphocytes (1.5-4 × 10^9 cells/L).

Table for Neutrophils (N=3 RCTs)

Author Year	Interventions & Comparators	Sample size	Preop value (mean)	Var. type	Var. – Preop	Follow-up value (mean)	Var. – Follow-up	Unit	Follow-up time (days)	p-value[a]
Yoko-yama 2016	Pre-op: Oral *Lactobacillus casei* strain Shirota + *Bifidobacterium breve* strain Yakult + galactooligosaccharides. Post-op: Enteral *Lactobacillus casei* strain Shirota + *Bifidobacterium breve* strain Yakult + galactooligosaccharides + EN.	22	3.1	range	1.3–5.5	7.8	4.3–15.5	10^3µl	3	p=0.699
	Pre-op: Standard care alone. Post-op: Enteral *Lactobacillus casei* strain Shirota + *Bifidobacterium breve* strain Yakult + galactooligosaccharides + EN.	22	3.5	range	1.7–5.8	7.2	3.7–16.8	10^3µl	3	
Yoko-yama 2014	Pre-op: oral or enteral *Lactobacillus casei* strain Shirota + *Bifidobacterium breve* strain Yakult + galactooligosaccharides.<br><br>Post-op: enteral *Lactobacillus casei* strain Shirota + *Bifidobacterium breve* strain Yakult + galactooligosaccharides + EN.	21	3100	NR	NR	3800	NR	µl	8	p<0.05
	Pre-op: standard care alone (ordinary diet).<br><br>Post-op: standard EN.	21	3900	NR	NR	4800	NR	µl	8	
Park 2020	*Bifidobacterium animalis* subsp. *lactis* HY8002 + *Lactobacillus casei* HY2782 + *L. plantarum* HY7712 + xylooligosaccharides + fructooligosaccharides	29	58.3	SD	8.16	52.76	9.72	NR	28	p>0.05
	Xylooligosaccharides + fructooligosaccharides	31	58.31	SD	11.74	57.5	7.96	NR	28	

[a]p-value between groups.

Table for C-reactive protein (N=13 RCTs)

Author Year	Interventions & Comparators	Sample size	Preop value (mean)	Var. type	Var. – Preop	Follow-up value (mean)	Var. – Follow-up	Unit	Follow-up time (days)	p-value[a]
Anderson 2004	*Lactobacillus acidophilus* La5 + *L. bulgaricus* + *Bifidobacterium lactis* Bb-12 + *Streptococcus thermophiles* capsule + oligofructose powder	49	5	IQR	0-50	65	10-125	mg/ml	7	p>0.05
	Placebo capsule + sucrose placebo powder	39	5	IQR	0-40	50	10-125	mg/ml	7	
Horvat 2010	*Pediacoccus pentosaceus* 5-33:3 + *Leuconostoc mesenteroides* 32–77:1 + *Lactobacillus paracasei* subsp. *paracasei* 19 + *L. plantarum* 2362 + betaglucan + inulin + pectin + resistant starch fibers [without mechanical bowel preparation]	20	17	range	1-93	111	2-262	mg/l	4	p=0.51
	Heat-inactivated lactobacilli + betaglucan + inulin + pectin + resistant starch fibers [without mechanical bowel preparation]	28	17	range	1-150	97	16-197	mg/l	4	
	Mechanical bowel preparation control	20	61	range	1-47	85	24–203	mg/l	4	
Kanazawa 2005	*Lactobacillus casei* strain Shirota + *Bifidobacterium breve* strain Yakult + galactooligosaccharides + EN + PN	21	1.5	SD	2.1	2.6	2.8	g/dl	14	p>0.05
	Standard EN + PN	23	2.1	SD	4	3.6	2.7	g/dl	14	
Krebs 2016	*Pediacoccus pentosaceus* 5-33:3 + *Leuconostoc mesenteroides* 32–77:1 + *Lactobacillus paracasei* subsp. *paracasei* 19 + *L. plantarum* 2362 + betaglucan + inulin + pectin + resistant starch fibers [without mechanical bowel preparation]	18	2[b]	range	0-117	104[b]	27-262	NR	3	p>0.05
	Betaglucan + inulin + pectin + resistant starch fibers [without mechanical bowel preparation]	20	4[b]	range	1-150	103[b]	16-239	NR	3	
	Mechanical bowel preparation control	16	3[b]	range	1.0-47	88[b]	24-177	NR	3	

Lages 2018	*Lactobacillus paracasei* LPC-31 + *L. rhamnosus* HN00l + *L. acidophilus* NCFM + *Bifidobacterium lactis* HN019 + fructooligosaccharides + standard EN	18	3038.1	SD	4847.6	10485.7	6676.2	nmol/l	8	p>0.05
	Maltodextrin placebo + standard EN	18	2933.3	SD	3695.2	13571.4	9590.4	nmol/l	8	
McNaught 2002	*Lactobacillus plantarum* 299v in an oatmeal-based drink	53	10	IQR	5.0-30	90	40-145	mg/l	16	p>0.05
	Standard care alone	65	8	IQR	5.0-30	70	35-100	mg/l	16	
Polakowski 2019	*Lactobacillus acidophilus* NCFM + *L. rhamnosus* HN001 + *L. paracasei* LPC-37 + *Bifidobacterium lactis* HN019 + fructooligosaccharides	36	10.0	SD	5.2	7.17	3.2	mg/dl	8	NR
	Maltodextrin placebo	37	10.6	SD	6.18	10.4	6.1	mg/dl	8	
Rayes 2002	Live *Lactobacillus plantarum* 299 + oat fiber + EN	30	1.1	SE	0.2	3.0	0.4	mg/dL	12	p>0.05
	Heat-killed *Lactobacillus plantarum* 299 + oat fiber + EN	30	2.1	SE	0.9	4.9	1.4	mg/dL	12	
	Standard total parenteral nutrition or fiber-free EN control	30	1.9	SE	0.8	4.3	0.8	mg/dL	12	
Sugawara 2006	Pre-op: Oral *Lactobacillus casei* strain Shirota + *Bifidobacterium breve* strain Yakult + galactooligosaccharides.<br>Post-op: Enteral *Lactobacillus casei* strain Shirota + *Bifidobacterium breve* strain Yakult + galactooligosaccharides + standard EN + PN.	41	1.5	NR	NR	0.8	NR	mg/dl	14	p>0.05
	Pre-op: Standard care alone.<br>Post-op: Enteral *Lactobacillus casei* strain Shirota + *Bifidobacterium breve* strain Yakult + galactooligosaccharides + standard EN + PN.	40	2.0	NR	NR	1.0	NR	mg/dl	14	
Usami 2011	*Lactobacillus casei* strain Shirota + *Bifidobacterium breve* strain Yakult + galactooligosaccharides [+ PN for 4 days post-op]	32	0.1	SD	0.1	1.3	1.1	mg/dL	16	p>0.05
	Standard care alone [+ PN for 4 days post-op]	29	0.2	SD	0.3	1.9	1.8	mg/dL	16	
Xu 2019	Bifidus-triple viable preparation + glucose solution	30	6.5	SD	0.6	20.2	4.4	mg/l	8	p<0.05

	Glucose solution	30	6.3	SD	0.4	46.7	5.3	mg/l	8	
Yokoyama 2014	Pre-op: oral or enteral *Lactobacillus casei* strain Shirota + *Bifidobacterium breve* strain Yakult + galactooligosaccharides.<br><br>Post-op: enteral *Lactobacillus casei* strain Shirota + *Bifidobacterium breve* strain Yakult + galactooligosaccharides + EN.	21	0.2	NR	NR	7	NR	mg/dl	8	p>0.05
	Pre-op: standard care alone (ordinary diet).<br><br>Post-op: standard EN.	21	0.3	NR	NR	7	NR	mg/dl	8	
Yokoyama 2016	Pre-op: Oral *Lactobacillus casei* strain Shirota + *Bifidobacterium breve* strain Yakult + galactooligosaccharides.<br>Post-op: Enteral *Lactobacillus casei* strain Shirota + *Bifidobacterium breve* strain Yakult + galactooligosaccharides + EN.	22	0.08	range	0.01-1.08	9.91	4.9-12.09	mg/dl	3	p=0.405
	Pre-op: Standard care alone.<br>Post-op: Enteral *Lactobacillus casei* strain Shirota + *Bifidobacterium breve* strain Yakult + galactooligosaccharides + EN.	22	0.12	range	0.01-2.77	8.42	3.58-15.22	mg/dl	3	

[a]p-value between groups.
[b]Median value was reported.

Table for Hospital Length of Stay (N=22 RCTs)

Author Year	Interventions & Comparators	Sample size	Length of Stay (days)[a]	Var. type	Var. value	p-value[b]
Anderson 2004	*Lactobacillus acidophilus* La5 + *L. bulgaricus* + *Bifidobacterium lactis* Bb-12 + *Streptococcus thermophiles* capsule + oligofructose powder	72	8	NR	NR	p>0.05
	Placebo capsule + sucrose placebo powder	65	8	NR	NR	
Consoli 2016	*Saccharomyces boulardii*	15	10	NR	NR	p>0.05
	Standard care alone	18	11	NR	NR	
Flesch 2017	*Lactobacillus acidophilus* NCFM + *L. rhamnosus* HN001 + *L. paracasei* LPC-37 + *Bifidobacterium lactis* HN019 + fructooligosaccharides	49	11.20	NR	NR	p>0.05
	Maltodextrin placebo	42	12.69	NR	NR	
Franko 2019	*Bifidobacterium breve* + *B. longum* + *B. infantis* + *Lactobacillus acidophilus* + *L. plantarum* + *L. paracasei* + *L. bulgaricus* + *Streptococcus thermophilus*	67	6.3	SD	9.1	p=0.421
	Placebo	68	5.2	SD	5.9	
Horvat 2010	*Pediacoccus pentosaceus* 5-33:3 + *Leuconostoc mesenteroides* 32–77:1 + *Lactobacillus paracasei* subsp. *paracasei* 19 + *L. plantarum* 2362 + betaglucan + inulin + pectin + resistant starch fibers [without mechanical bowel preparation]	20	10.95	NR	NR	p>0.05
	Heat-inactivated lactobacilli + betaglucan + inulin + pectin + resistant starch fibers [without mechanical bowel preparation]	28	9.20	NR	NR	
	Mechanical bowel preparation control	20	9.50	NR	NR	
Kanazawa 2005	*Lactobacillus casei* strain Shirota + *Bifidobacterium breve* strain Yakult + galactooligosaccharides + EN + PN	21	36.9	SD	16.4	p=0.069
	Standard EN + PN	23	47.0	SD	19.4	
Kotzampassi 2015	*Lactobacillus acidophilus* LA-5 + *L. plantarum* + *Bifidobacterium lactis* BB-12 + *Saccharomyces boulardii*	84	8	NR	NR	NR
	Glucose polymer placebo	80	10	NR	NR	
Krebs 2016	*Pediacoccus pentosaceus* 5-33:3 + *Leuconostoc mesenteroides* 32–77:1 + *Lactobacillus paracasei* subsp. *paracasei* 19 + *L. plantarum* 2362 + betaglucan + inulin + pectin + resistant starch fibers [without mechanical bowel preparation]	18	10.2	NR	NR	p>0.05

	Betaglucan + inulin + pectin + resistant starch fibers [without mechanical bowel preparation]	20	11.3	NR	NR	
	Mechanical bowel preparation control	16	10.5	NR	NR	
Lages 2018	*Lactobacillus paracasei* LPC-31 + *L. rhamnosus* HN00l + *L. acidophilus* NCFM + *Bifidobacterium lactis* HN019 + fructooligosaccharides + standard EN	18	10.5	range	3-90	p>0.05
	Maltodextrin placebo + standard EN	18	9.0	range	5-21	
Liu 2015	*Lactobacillus plantarum* CGMCC No. 1258 + *L. acidophilus* LA-11 + *Bifidobacterium longum* BL-88	66	11.26	SD	2.52	p<0.001
	Maltodextrin placebo	68	12.96	SD	3.06	
Nomura 2007	*Enterococcus fecalis* T-110 + *Clostridium butyricum* TO-A + *Bacillus mesentericus* TO-A	30	19	range	11-40	p=0.04
	Standard care alone	34	24	range	11-91	
Polakowski 2019	*Lactobacillus acidophilus* NCFM + *L. rhamnosus* HN001 + *L. paracasei* LPC-37 + *Bifidobacterium lactis* HN019 + fructooligosaccharides	36	3	range	3-5	p<0.001
	Maltodextrin placebo	37	4	range	3-21	
Rayes 2002	Live *Lactobacillus plantarum* 299 + oat fiber + EN	30	14	SE	4.0	p>0.05
	Heat-killed *Lactobacillus plantarum* 299 + oat fiber + EN	30	15	SE	7.4	
	Standard total parenteral nutrition or fiber-free EN control	30	16	SE	5.5	
Rayes 2007	*Pediacoccus pentosaceus* 5-33:3 + *Leuconostoc mesenteroides* 32–77:1 + *Lactobacillus paracasei* subsp. *paracasei* 19 + *L. plantarum* 2362 + betaglucan + inulin + pectin + resistant starch fibers + EN	40	17	SE	8	p>0.05
	Betaglucan + inulin + pectin + resistant starch fibers + EN	40	22	SE	16	
Sommacal 2015	*Lactobacillus acidophilus* 10 + *L. rhamnosus* HS 111 + *L. casei* 10 + *Bifidobacterium bifidum* + fructooligosaccharides	23	12	SD	5	p=0.008
	Sucrose placebo	23	23	SD	14	
Tan 2016	*Lactobacillus acidophilus* BCMC12130 + *L. casei* BCMC12313 + *L. lactis* BCMC12451 + *Bifidobacterium bifidum* BCMC02290 + *B. longum* BCMC02120 + *B. infantis* BCMC02129	20	6.5	range	4-30	p=0.012
	Placebo	20	13.0	range	5-25	

Usami 2011	*Lactobacillus casei* strain Shirota + *Bifidobacterium breve* strain Yakult + galactooligosaccharides [+ PN for 4 days post-op]	32	18.5	SD	3.2	p>0.05
	Standard care alone [+ PN for 4 days post-op]	29	20.3	SD	4.2	
Yang 2016	*Bifidobacterium longum* + *Lactobacillus acidophilus* + *Enterococcus faecalis*	30	15.9	SD	4.92	p=0.487
	Maltodextrin + sucrose placebo	30	15.0	SD	4.31	
Yokoyama 2014	Pre-op: oral or enteral *Lactobacillus casei* strain Shirota + *Bifidobacterium breve* strain Yakult + galactooligosaccharides.<br><br>Post-op: enteral *Lactobacillus casei* strain Shirota + *Bifidobacterium breve* strain Yakult + galactooligosaccharides + EN.	21	32	range	19-85	p=0.676
	Pre-op: standard care alone (ordinary diet).<br><br>Post-op: standard EN.	21	23	range	15-86	
Yokoyama 2016	Pre-op: Oral *Lactobacillus casei* strain Shirota + *Bifidobacterium breve* strain Yakult + galactooligosaccharides.<br>Post-op: Enteral *Lactobacillus casei* strain Shirota + *Bifidobacterium breve* strain Yakult + galactooligosaccharides + EN.	22	37	range	16-103	p=0.681
	Pre-op: Standard care alone.<br>Post-op: Enteral *Lactobacillus casei* strain Shirota + *Bifidobacterium breve* strain Yakult + galactooligosaccharides + EN.	22	35	range	13-59	
Zhang 2012	*Bifidobacterium longum* + *Lactobacillus acidophilus* + *Enterococcus faecalis*	30	12	SD	3	p=0.109
	Maltodextrin placebo	30	14	SD	3	
Zhao 2017	*Bifidobacterium* + *Lactobacillus* + fiber + EN	40	7.42	SD	0.83	p<0.001[c]
	Fiber-free EN control	40	8.05	SD	0.61	
	Fiber-enriched EN	40	7.61	SD	0.72	

[a]Mean or median value as reported in the studies.
[b]p-value between groups.
[c]p-value for probiotics group compared to fiber-free enteral nutrition control.

Table for Pain (N=7 RCTs)

Author Year	Interventions & Comparators	Sample size	Preop score	Postop value	Follow-up time (days)	Name of outcome	p-value[a]
Franko 2019	*Bifidobacterium breve* + *B. longum* + *B. infantis* + *Lactobacillus acidophilus* + *L. plantarum* + *L. paracasei* + *L. bulgaricus* + *Streptococcus thermophilus*	67	1.6[b]	1.25[b]	30	Pain score[c] GP2 (FACT-G7 subscale)	p>0.05
	Placebo	68	1.2[b]	1.5[b]	30	Pain score[c] GP2 (FACT-G7 subscale)	
Lages 2018	*Lactobacillus paracasei* LPC-31 + *L. rhamnosus* HN00l + *L. acidophilus* NCFM + *Bifidobacterium lactis* HN019 + fructooligosaccharides + standard EN	18	NA	2	7	Number of patients with abdominal cramps	p>0.05
	Maltodextrin placebo + standard EN	18	NA	2	7	Number of patients with abdominal cramps	
Liu 2015	*Lactobacillus plantarum* CGMCC No. 1258 + *L. acidophilus* LA-11 + *Bifidobacterium longum* BL-88	66	NA	15	30	Number of patients with abdominal cramping	p=0.017
	Maltodextrin placebo	68	NA	33	30	Number of patients with abdominal cramping	
Rayes 2002	Live *Lactobacillus plantarum* 299 + oat fiber + EN	30	NA	4	45	Abdominal cramps	NR
	Heat-killed *Lactobacillus plantarum* 299 + oat fiber + EN	30	NA	5	45	Abdominal cramps	
	Standard total parenteral nutrition or fiber-free EN control	30	NA	6	45	Abdominal cramps	
Rayes 2007	*Pediacoccus pentosaceus* 5-33:3 + *Leuconostoc mesenteroides* 32–77:1 + *Lactobacillus paracasei* subsp. *paracasei* 19 + *L. plantarum* 2362 + betaglucan + inulin + pectin + resistant starch fibers + EN	40	NA	3	30	Abdominal cramps from EN intervention	NR
	Betaglucan + inulin + pectin + resistant starch fibers + EN	40	NA	6	30	Abdominal cramps from EN intervention	

Rayes 2012	*Pediacoccus pentosaceus* 5-33:3 + *Leuconostoc mesenteroides* 32–77:1 + *Lactobacillus paracasei* subsp. *paracasei* 19 + *L. plantarum* 2362 + betaglucan + inulin + pectin + resistant starch fibers + EN	9	NA	3	NR	Abdominal distension and cramps	p>0.05
	Betaglucan + inulin + pectin + resistant starch fibers + EN	10	NA	3	NR	Abdominal distension and cramps	
Zhao 2017	*Bifidobacterium* + *Lactobacillus* + fiber + EN	40	NA	1	7	Number of patients with abdominal pain	p>0.05
	Fiber-enriched EN	40	NA	1	7	Number of patients with abdominal pain	
	Fiber-free EN control	40	NA	2	7	Number of patients with abdominal pain	

[a]p-value between groups.
[b]Variance was not reported.
[c]Higher score represents worse pain; scale range 0-4.

Table for Blood Loss (N=11 RCTs)

Author Year	Interventions & Comparators	Sample size	Blood loss (ml)[a]	Var. type	Var. value	p-value[b]
Komatsu 2016	*Lactobacillus casei* strain Shirota + *Bifidobacterium breve* strain Yakult + galactooligosaccharides	168	65[c]	NR	NR	NR
	Standard care alone	194	57[c]	NR	NR	
Liu 2015	*Lactobacillus plantarum* CGMCC No. 1258 + *L. acidophilus* LA-11 + *Bifidobacterium longum* BL-88	66	352.56	SD	169.26	NR
	Maltodextrin placebo	68	336.26	SD	182.68	
Mangell 2012	*Lactobacillus plantarum* 299v in an oatmeal-based drink	32	300	NR	NR	NR
	Oatmeal-based placebo drink without probiotics	32	300	NR	NR	
Nomura 2007	*Enterococcus fecalis* T-110 + *Clostridium butyricum* TO-A + *Bacillus mesentericus* TO-A	30	835	range	300-4700	p=0.30
	Standard care alone	34	900	range	500-3780	
Okazaki 2013	*Lactobacillus casei* strain Shirota + *Bifidobacterium breve* strain Yakult + galactooligosaccharides	25	140	range	5-650	p>0.05
	Standard care alone	23	230	range	5-1300	
Sadahiro 2014	*Bifidobacterium bifidum* + maltooligosaccharide [plus single IV dose of flomoxef; & standard mechanical bowel preparation]	100	141	SD	154	p=0.77
	Standard care alone control [plus single IV dose of flomoxef; & standard mechanical bowel preparation. No probiotic or oral antibiotics]	95	155	SD	211	
	Kanamycin sulfate + metronidazole [plus single IV dose of flomoxef; & standard mechanical bowel preparation]	99	147	SD	140	
Sugawara 2006	Pre-op: Oral *Lactobacillus casei* strain Shirota + *Bifidobacterium breve* strain Yakult + galactooligosaccharides.	41	1761	SD	831	NR
	Pre-op: Standard care alone.	40	1972	SD	769	
Usami 2011	*Lactobacillus casei* strain Shirota + *Bifidobacterium breve* strain Yakult + galactooligosaccharides [+ PN for 4 days post-op]	32	1111.9	SD	747.3	p<0.05
	Standard care alone [+ PN for 4 days post-op]	29	695.2	SD	433.1	

Yang 2016	*Bifidobacterium longum* + *Lactobacillus acidophilus* + *Enterococcus faecalis*	30	123.4	SD	30.5	p=0.388
	Maltodextrin + sucrose placebo	30	116.6	SD	29.79	
Yokoyama 2014	Pre-op: oral or enteral *Lactobacillus casei* strain Shirota + *Bifidobacterium breve* strain Yakult + galactooligosaccharides.	21	1119	range	412-6698	p=0.352
	Pre-op: standard care alone (ordinary diet).	21	977	range	324-2567	
Yokoyama 2016	Pre-op: Oral *Lactobacillus casei* strain Shirota + *Bifidobacterium breve* strain Yakult + galactooligosaccharides.	22	887	range	562-2282	p=0.296
	Pre-op: Standard care alone.	22	982	range	188-5609	

[a]Mean or median value as reported in the studies.

[b]p-value between groups.

[c]Estimated converted value using 1.05 g/ml.

Table S5. Adverse Events and Other Complications in 36 RCTs

Author Year	Interventions & Comparators	Sample size	Adverse Event or Other Complication #1- Name	AE or OC #1- No.	AE or OC #2- Name	AE or OC #2- No.
Anderson 2004	*Lactobacillus acidophilus* La5 + *L. bulgaricus* + *Bifidobacterium lactis* Bb-12 + *Streptococcus thermophiles* capsule + oligofructose powder	72	Unpalatable (wrt oligofructose powder; continued with just the probiotic capsule)	1		
Anderson 2004	Placebo capsule + sucrose placebo powder	65	Unpalatable intervention	0		
Aso 1992	*Lactobacillus casei*	25	Adverse reactions	0		
Aso 1992	Standard care alone	25	NA	NA		
Cho 2019	*Lactobacillus plantarum* CJLP243	17	NR			
Cho 2019	Maltodextrin + glucose placebo	19	NR			
Consoli 2016	*Saccharomyces boulardii*	15	Adverse effects reported with the use of probiotics	0	Noninfectious complications requiring re-laparotomy	1
Consoli 2016	Standard care alone	18	NA	NA	Noninfectious complications requiring re-laparotomy	1
Diepenhorst 2011	*Bifidobacterium bifidum* + *B. infantis* + *Lactobacillus acidophilus* + *L. casei* + *L. salivarius* + *L. lactis*	10	Noninfectious complications	4		
Diepenhorst 2011	Selective decontamination of the digestive tract (SDD antibiotics regimen)	10	Noninfectious complications	2		
Diepenhorst 2011	Standard tx control [neither probiotics nor SDD]	10	Noninfectious complications	4		
Flesch 2017	*Lactobacillus acidophilus* NCFM + *L. rhamnosus* HN001 + *L. paracasei* LPC-37 + *Bifidobacterium lactis* HN019 + fructooligosaccharides	49	noninfectious complications	p= 0.161	re-hospitalization rates	p> 0.05

Flesch 2017	Maltodextrin placebo	42	NA	NA		
Franko 2019	*Bifidobacterium breve* + *B. longum* + *B. infantis* + *Lactobacillus acidophilus* + *L. plantarum* + *L. paracasei* + *L. bulgaricus* + *Streptococcus thermophilus*	67	30-day readmission	11	Discontinuation of the intervention due to severe complication	0
Franko 2019	Placebo	68	30-day readmission	3	Discontinuation of the intervention due to severe complication	1
Horvat 2010	*Pediacoccus pentosaceus* 5-33:3 + *Leuconostoc mesenteroides* 32–77:1 + *Lactobacillus paracasei* subsp. *paracasei* 19 + *L. plantarum* 2362 + betaglucan + inulin + pectin + resistant starch fibers [without mechanical bowel preparation]	20	Major complications	0		
Horvat 2010	Heat-inactivated lactobacilli + betaglucan + inulin + pectin + resistant starch fibers [without mechanical bowel preparation	28	Major complications	0		
Horvat 2010	Mechanical bowel preparation control	20	Major complications	0		
Kanazawa 2005	*Lactobacillus casei* strain Shirota + *Bifidobacterium breve* strain Yakult + galactooligosaccharides + EN + PN	21	NR			
Kanazawa 2005	Standard EN + PN	23	NR			
Komatsu 2016	*Lactobacillus casei* strain Shirota + *Bifidobacterium breve* strain Yakult + galactooligosaccharides	168	Adverse effects of synbiotics	0	Readmission	2
Komatsu 2016	Standard care alone	194	NA	NA	Readmission	1
Kotzampassi 2015	*Lactobacillus acidophilus* LA-5 + *L. plantarum* + *Bifidobacterium lactis* BB-12 + *Saccharomyces boulardii*	84	Noninfectious complications	14		
Kotzampassi 2015	Glucose polymer placebo	80	Noninfectious complications	17		
Krebs 2016	*Pediacoccus pentosaceus* 5-33:3 + *Leuconostoc mesenteroides* 32–77:1 + *Lactobacillus paracasei* subsp. *paracasei* 19 + *L. plantarum* 2362 + betaglucan + inulin + pectin + resistant starch fibers [without mechanical bowel preparation]	18	NR			

Krebs 2016	Betaglucan + inulin + pectin + resistant starch fibers [without mechanical bowel preparation]	20	NR			
Krebs 2016	Mechanical bowel preparation control	16	NR			
Lages 2018	*Lactobacillus paracasei* LPC-31 + *L. rhamnosus* HN00l + *L. acidophilus* NCFM + *Bifidobacterium lactis* HN019 + fructooligosaccharides + standard EN	18	Flatulence	5	Abdominal bloating	1
Lages 2018	Maltodextrin placebo + standard EN	18	Flatulence	5	Abdominal bloating	3
Lages 2018	*Lactobacillus paracasei* LPC-31 + *L. rhamnosus* HN00l + *L. acidophilus* NCFM + *Bifidobacterium lactis* HN019 + fructooligosaccharides + standard EN	18	Noninfectious complications	1		
Lages 2018	Maltodextrin placebo + standard EN	18	Noninfectious complications	2		
Liu 2015	*Lactobacillus plantarum* CGMCC No. 1258 + *L. acidophilus* LA-11 + *Bifidobacterium longum* BL-88	66	Side effects of probiotics	0		
Liu 2015	Maltodextrin placebo	68	NA	NA		
Mangell 2012	*Lactobacillus plantarum* 299v in an oatmeal-based drink	32	Adverse effects	0		
Mangell 2012	Oatmeal-based placebo drink without probiotics	32	NA	NA		
McNaught 2002	*Lactobacillus plantarum* 299v in an oatmeal-based drink	64	paralytic ileus	12	nausea	16
McNaught 2002	Standard care alone	65	NR	NR	NR	NR
McNaught 2002	*Lactobacillus plantarum* 299v in an oatmeal-based drink	64	Disliked taste affecting compliance	19		
McNaught 2002	Standard care alone	65	NR	NR		
Nomura 2007	*Enterococcus fecalis* T-110 + *Clostridium butyricum* TO-A + *Bacillus mesentericus* TO-A	30	Delayed gastric emptying	3		
Nomura 2007	Standard care alone	34	Delayed gastric emptying	7		
Okazaki 2013	*Lactobacillus casei* strain Shirota + *Bifidobacterium breve* strain Yakult + galactooligosaccharides	25	Problems related to synbiotics	0	Bile spillage	0
Okazaki	Standard care alone	23	NA	NA	Bile spillage	1

2013						
Park 2020	*Bifidobacterium animalis* subsp. *lactis* HY8002 + *Lactobacillus casei* HY2782 + *L. plantarum* HY7712 + xylooligosaccharides + fructooligosaccharides	33	Severe adverse events (MEDRA v21.0)	0	Patients with adverse events (hepatobiliary disorder was the most common)	11
Park 2020	Xylooligosaccharides + fructooligosaccharides	35	Severe adverse events (MEDRA v21.0)	2	Patients with adverse events (GI discomfort was the most common)	16
Park 2020	*Bifidobacterium animalis* subsp. *lactis* HY8002 + *Lactobacillus casei* HY2782 + *L. plantarum* HY7712 + xylooligosaccharides + fructooligosaccharides	33	Discontinued intervention	1	Noninfectious complications (Clavien-Dindo classification ≥2)	0
Park 2020	Xylooligosaccharides + fructooligosaccharides	35	Discontinued intervention	0	Noninfectious complications (Clavien-Dindo classification ≥2)	4
Park 2020	*Bifidobacterium animalis* subsp. *lactis* HY8002 + *Lactobacillus casei* HY2782 + *L. plantarum* HY7712 + xylooligosaccharides + fructooligosaccharides	33	AEs where a relationship with the test powder could not be excluded (cholelithiasis and tremor)	2		
Park 2020	Xylooligosaccharides + fructooligosaccharides	35	AEs where a relationship with the test powder could not be excluded (diarrhea, tremor and abnormal liver function)	3		
Polakowski	*Lactobacillus acidophilus* NCFM + *L. rhamnosus* HN001 + *L.*	36	Flatulence (most	4	Noninfectious	0

2019	*paracasei* LPC-37 + *Bifidobacterium lactis* HN019 + fructooligosaccharides		common side-effect with synbiotics; "synbiotics were well tolerated")		complications	
Polakowski 2019	Maltodextrin placebo	37	Flatulence	3	Noninfectious complications	4
Rayes 2002	Live *Lactobacillus plantarum* 299 + oat fiber + EN	30	Abdominal distention	3	Intolerance of EN intervention	0
Rayes 2002	Heat-killed *Lactobacillus plantarum* 299 + oat fiber + EN	30	Abdominal distention	6	Intolerance of EN intervention	0
Rayes 2002	Standard total parenteral nutrition or fiber-free EN control	30	Abdominal distention	4	Intolerance of TPN intervention	0
Rayes 2002	Live *Lactobacillus plantarum* 299 + oat fiber + EN	30	Noninfectious complications	4		
Rayes 2002	Heat-killed *Lactobacillus plantarum* 299 + oat fiber + EN	30	Noninfectious complications	7		
Rayes 2002	Standard total parenteral nutrition or fiber-free EN control	30	Noninfectious complications	9		
Rayes 2007	*Pediacoccus pentosaceus* 5-33:3 + *Leuconostoc mesenteroides* 32–77:1 + *Lactobacillus paracasei* subsp. *paracasei* 19 + *L. plantarum* 2362 + betaglucan + inulin + pectin + resistant starch fibers + EN	40	Noninfectious complications	9	Intolerance of synbiotics	0
Rayes 2007	Betaglucan + inulin + pectin + resistant starch fibers + EN	40	Noninfectious complications	10	NA	NA
Rayes 2012	*Pediacoccus pentosaceus* 5-33:3 + *Leuconostoc mesenteroides* 32–77:1 + *Lactobacillus paracasei* subsp. *paracasei* 19 + *L. plantarum* 2362 + betaglucan + inulin + pectin + resistant starch fibers + EN	9	Noninfectious complications	2	Intolerance of synbiotics	0
Rayes 2012	Betaglucan + inulin + pectin + resistant starch fibers + EN	10	Noninfectious complications	3	NA	NA
Reddy 2007	*Lactobacillus acidophilus* La5 + *L. bulgaricus* + *Bifidobacterium lactis* Bb-12 + *Streptococcus thermophilus* + oligofructose + neomycin + mechanical bowel preparation	20	NR			
Reddy 2007	*Lactobacillus acidophilus* La5 + *L. bulgaricus* + *Bifidobacterium lactis* Bb-12 + *Streptococcus thermophilus* + oligofructose + neomycin [without mechanical bowel preparation]	22	NR			

Reddy 2007	Neomycin + mechanical bowel preparation control	22	NR			
Reddy 2007	Mechanical bowel preparation only	24	NR			
Sadahiro 2014	*Bifidobacterium bifidum* + maltooligosaccharide [plus single IV dose of flomoxef; & standard mechanical bowel preparation]	100	NR			
Sadahiro 2014	Kanamycin sulfate + metronidazole [plus single IV dose of flomoxef; & standard mechanical bowel preparation]	99	NR			
Sadahiro 2014	Standard care alone control [plus single IV dose of flomoxef; & standard mechanical bowel preparation. No probiotic or oral antibiotics]	95	NR			
Sommacal 2015	*Lactobacillus acidophilus* 10 + *L. rhamnosus* HS 111 + *L. casei* 10 + *Bifidobacterium bifidum* + fructooligosaccharides	23	Noninfectious complications	6		
Sommacal 2015	Sucrose placebo	23	Noninfectious complications	14		
Sugawara 2006	Pre-op: Oral *Lactobacillus casei* strain Shirota + *Bifidobacterium breve* strain Yakult + galactooligosaccharides.<br>Post-op: Enteral *Lactobacillus casei* strain Shirota + *Bifidobacterium breve* strain Yakult + galactooligosaccharides + standard EN + PN.	41	Problems related to synbiotics	0		
Sugawara 2006	Pre-op: Standard care alone.<br>Post-op: Enteral *Lactobacillus casei* strain Shirota + *Bifidobacterium breve* strain Yakult + galactooligosaccharides + standard EN + PN.	40	NA	NA		
Tan 2016	*Lactobacillus acidophilus* BCMC12130 + *L. casei* BCMC12313 + *L. lactis* BCMC12451 + *Bifidobacterium bifidum* BCMC02290 + *B. longum* BCMC02120 + *B. infantis* BCMC02129	20	Noninfectious complications	1		
Tan 2016	Placebo	20	Noninfectious complications	4		
Usami 2011	*Lactobacillus casei* strain Shirota + *Bifidobacterium breve* strain Yakult + galactooligosaccharides [+ PN for 4 days post-op]	32	Adverse effects of synbiotics	0	Noninfectious complications	4
Usami 2011	Standard care alone [+ PN for 4 days post-op]	29	NA	NA	Noninfectious complications	3
Xu 2019	Bifidus-triple viable preparation + glucose solution	30	NR			
Xu 2019	Glucose solution	30	NR			
Yang 2016	*Bifidobacterium longum* + *Lactobacillus acidophilus* + *Enterococcus faecalis*	30	Side-effect of drug	0	Abdominal distension	9

Yang 2016	Maltodextrin + sucrose placebo	30	Side-effect of drug	0	Abdominal distension	13
Yokoyama 2014	Pre-op: oral or enteral *Lactobacillus casei* strain Shirota + *Bifidobacterium breve* strain Yakult + galactooligosaccharides.<br><br>Post-op: enteral *Lactobacillus casei* strain Shirota + *Bifidobacterium breve* strain Yakult + galactooligosaccharides + EN.	21	Noninfectious complications	11		
Yokoyama 2014	Pre-op: standard care alone (ordinary diet).<br><br>Post-op: standard EN.	21	Noninfectious complications	10		
Yokoyama 2016	Pre-op: Oral *Lactobacillus casei* strain Shirota + *Bifidobacterium breve* strain Yakult + galactooligosaccharides.<br>Post-op: Enteral *Lactobacillus casei* strain Shirota + *Bifidobacterium breve* strain Yakult + galactooligosaccharides + EN.	22	Pancreatic fistula ≥ grade B	8		
Yokoyama 2016	Pre-op: Standard care alone.<br>Post-op: Enteral *Lactobacillus casei* strain Shirota + *Bifidobacterium breve* strain Yakult + galactooligosaccharides + EN.	22	Pancreatic fistula ≥ grade B	3		
Zhang 2012	*Bifidobacterium longum* + *Lactobacillus acidophilus* + *Enterococcus faecalis*	30	NR			
Zhang 2012	Maltodextrin placebo	30	NR			
Zhao 2017	*Bifidobacterium* + *Lactobacillus* + fiber + EN	40	Excluded patients due to severe complications or EN intolerance	0	Abdominal distension	1
Zhao 2017	Fiber-enriched EN	40	Excluded patients due to severe complications or EN intolerance	0	Abdominal distension	2
Zhao 2017	Fiber-free EN control	40	Excluded patients due to severe complications or EN intolerance	0	Abdominal distension	3

Zheng 2019	*Bifidobacterium infantis* + *Lactobacillus acidophilus* + *Enterococcus faecalis* + *Bacillus cereus*	50	NR			
Zheng 2019	Placebo	50	NR			

Tables S6. Results from Nonrandomized & Observational Studies

Alphabetical List of the Included Nonrandomized & Observational Studies

NB: The number below corresponds to the reference number cited in the main report's References section:

72. Aisu N, Tanimura S, Yamashita Y, et al. Impact of perioperative probiotic treatment for surgical site infections in patients with colorectal cancer. *Exp Ther Med.* 2015;10(3):966-972.
73. Ding Y, Gao Z, Sun Z, et al. Enhanced recovery program in liver resection surgery: a single center experience. *Translational Cancer Research; Vol 7, No 4 (August 2018): Translational Cancer Research.* 2018.
74. Fujio A, Miyagi S, Tokodai K, et al. Effects of a new perioperative enhanced recovery after surgery protocol in hepatectomy for hepatocellular carcinoma. *Surg Today.* 2020;50(6):615-622.
75. Mao J, Zhang SZ, Du P, et al. Probiotics Can Boost the Antitumor Immunity of CD8(+)T Cells in BALB/c Mice and Patients with Colorectal Carcinoma. *J Immunol Res.* 2020;2020:4092472.
76. Mizuta M, Endo I, Yamamoto S, et al. Perioperative supplementation with bifidobacteria improves postoperative nutritional recovery, inflammatory response, and fecal microbiota in patients undergoing colorectal surgery: a prospective, randomized clinical trial. *Biosci Microbiota Food Health.* 2016;35(2):77-87.
77. Rifatbegovic Z, Mesic D, Ljuca F, et al. Effect of probiotics on liver function after surgery resection for malignancy in the liver cirrhotic. *Med Arh.* 2010;64(4):208-211.

Table of Nonrandomized or Cohort Studies for Mortality

Author Year	Tx Name	Sample size	No. deaths	Follow-up time
Fujio 2020	ERAS periop program (with Synbiotics)	42	0	NR
	Pre-ERAS periop program	55	0	NR
Rifatbegovic 2010	Synbiotics	60	8	1 year
	No added tx	60	20	1 year

Table of Nonrandomized or Cohort Studies for Progression Free Survival

Author Year	Tx Name	Sample size	PFS (median)
Mao 2020	*Bifidobacteria*	62	1531 days
	No added tx	60	1294 days

Table of Nonrandomized or Cohort Studies for Infections

Author Year	Tx Name	Sample size	No. infections	Follow-up time	Type of infection
Aisu 2015	BIO-THREE probiotics	75	5	30 days	enteritis
	No added tx	81	3	30 days	enteritis
	BIO-THREE probiotics	75	1	30 days	pneumonia
	No added tx	81	0	30 days	pneumonia
	BIO-THREE probiotics	75	7	30 days	surgical site infections (superficial or deep incisional/space/organ)
	No added tx	81	20	30 days	surgical site infections (superficial or deep incisional/space/organ)
	BIO-THREE probiotics	75	1	30 days	urinary tract infection
	No added tx	81	0	30 days	urinary tract infection
Fujio 2020	ERAS periop program (with Synbiotics)	42	0	NR	Grade IIIa (Clavien-Dindo) abdominal abscess
	Pre-ERAS periop program	55	1	NR	Grade IIIa (Clavien-Dindo) abdominal abscess
	ERAS periop program (with Synbiotics)	42	0	NR	Grade IIIa (Clavien-Dindo) wound infection
	Pre-ERAS periop program	55	1	NR	Grade IIIa (Clavien-Dindo) wound infection
	ERAS periop program (with Synbiotics)	42	0	NR	Sepsis
	Pre-ERAS periop program	55	1	NR	Sepsis
Mizuta 2016	*Bifidobacterium longum* BB536	23	2	14 days	Superficial surgical site infections
	No added tx	22	3	14 days	Superficial surgical site infections

Table of Nonrandomized or Cohort Studies for Additional Postop Antibiotic Treatment

Author Year	Tx Name	Sample size	No. of patients	Follow-up time
Mizuta 2016	*Bifidobacterium longum* BB536	23	5	14 days
	No added tx	22	2	14 days

Table of Nonrandomized or Cohort Studies for Hospital Length of Stay

Author Year	Tx Name	Sample size	Hospitalization length (days; mean/ median)	Variance type	Variance value
Ding 2018a (open)	*Clostridium butyricum* MIYAIRI + ERAS multidisciplinary periop program	11	8.55	SD	3.30
	ERAS multidisciplinary periop program	9	8.11	SD	3.92
Ding 2018b (lapar.)	*Clostridium butyricum* MIYAIRI + ERAS multidisciplinary periop program	27	5.63	SD	3.00
	ERAS multidisciplinary periop program	22	7.09	SD	4.20
Fujio 2020	ERAS periop program (with Synbiotics)	42	13	range	6-75
	Pre-ERAS periop program	55	18	range	8-46

Table of Nonrandomized or Cohort Studies for Pain

Author Year	Tx Name	Sample size	Follow-up value	Var. type	Var. value	Follow-up time	Name of outcome
Ding 2018a (open)	*Clostridium butyricum* MIYAIRI + ERAS multidisciplinary periop program	11	0.91	SD	1.22	1st postop day	Pain score[a]
	ERAS multidisciplinary periop program	9	0.67	SD	1.00	1st postop day	Pain score[a]
Ding 2018b (lapar.)	*Clostridium butyricum* MIYAIRI + ERAS multidisciplinary periop program	27	1.00	SD	1.07	1st postop day	Pain score[a]
	ERAS multidisciplinary periop program	22	0.55	SD	0.80	1st postop day	Pain score[a]

[a]Numerical rating scale range 1-10; preoperative values were not reported.

Table of Nonrandomized or Cohort Studies for Blood Loss

Author Year	Tx Name	Sample size	Blood loss (ml; mean/ median)	Variance type	Variance value
Aisu 2015	BIO-THREE probiotics	75	100	range	0-1190
Aisu 2015	No added tx	81	120	range	0-2060
Ding 2018a (open)	Clostridium butyricum MIYAIRI + ERAS multidisciplinary periop program	11	174.09	SD	136.69
Ding 2018a (open)	ERAS multidisciplinary periop program	9	144.44	SD	88.19
Ding 2018b (lapar.)	Clostridium butyricum MIYAIRI + ERAS multidisciplinary periop program	27	140.00	SD	123.03
Ding 2018b (lapar.)	ERAS multidisciplinary periop program	22	203.33	SD	196.88
Fujio 2020	ERAS periop program (with Synbiotics)	42	635	range	5-3585
Fujio 2020	Pre-ERAS periop program	55	895	range	25-11686

Table of All Nonrandomized/Cohort Studies for Adverse Events and Other Complications

Author Year	Tx Name	Sample size	Follow-up time	AE1 name	No. AE1	AE2 name	No. AE2	AE3 name	No. AE3	AE4 name	No. AE4
Aisu 2015	BIO-THREE probiotics	75	30 days	NR							
	No added tx	81	30 days	NR							
Ding 2018a (open)	*Clostridium butyricum* MIYAIRI + ERAS multidisciplinary periop program	11	30 days	NR							
	ERAS multidisciplinary periop program	9	30 days	NR							
Ding 2018b (lapar.)	*Clostridium butyricum* MIYAIRI + ERAS multidisciplinary periop program	27	30 days	NR							
	ERAS multidisciplinary periop program	22	30 days	NR							
Fujio 2020	ERAS periop program (with Synbiotics)	42	75 days	Grade[a] IIIb complication	2	Grade[a] IIIa complication	4	Grade[a] ≤ II complication	36	Unable to receive synbiotic due to diarrhea	1
	Pre-ERAS periop program	55	75 days	Grade[a] IIIb complication	0	Grade[a] IIIa complication	19	Grade[a] ≤ II complication	35	NR	NR
Mao	*Bifidobacteria*	62	5 years	NR							

2020	No added tx	60	5 years	NR							
Mizuta 2016	*Bifidobacterium longum* BB536	23	14 days	Anastomotic leak	3						
	No added tx	22	14 days	Anastomotic leak	1						
Rifatbeg-ovic 2010	Synbiotics	60	1 year	early postop complication	8	late postop complication	5				
	No added tx	60	1 year	early postop complication	20	late postop complication	12				

[a]According to Clavien-Dindo classification.
